# A Novel Long-Noncoding RNA LncZFAS1 Prevents MPP^+^-Induced Neuroinflammation Through MIB1 Activation

**DOI:** 10.1007/s12035-021-02619-z

**Published:** 2021-11-13

**Authors:** Ziman Zhu, Peiling Huang, Ruifeng Sun, Xiaoling Li, Wenshan Li, Weijun Gong

**Affiliations:** 1 Beijing Rehabilitation Medicine Academy, Capital Medical University, Beijing, 100144 China; 2 Department of Neurological Rehabilitation, Beijing Rehabilitation Hospital, Capital Medical University, Beijing, 100144 China

**Keywords:** LncZFAS, Parkinson’s disease, Pyroptosis, MIB1, TXNIP, NLRP3

## Abstract

Parkinson’s disease remains one of the leading neurodegenerative diseases in developed countries. Despite well-defined symptomology and pathology, the complexity of Parkinson’s disease prevents a full understanding of its etiological mechanism. Mechanistically, α-synuclein misfolding and aggregation appear to be central for disease progression, but mitochondrial dysfunction, dysfunctional protein clearance and ubiquitin/proteasome systems, and neuroinflammation have also been associated with Parkinson’s disease. Particularly, neuroinflammation, which was initially thought to be a side effect of Parkinson’s disease pathogenesis, has now been recognized as driver of Parkinson’s disease exacerbation. Next-generation sequencing has been used to identify a plethora of long noncoding RNAs (lncRNA) with important transcriptional regulatory functions. Moreover, a myriad of lncRNAs are known to be regulators of inflammatory signaling and neurodegenerative diseases, including IL-1β secretion and Parkinson’s disease. Here, LncZFAS1 was identified as a regulator of inflammasome activation, and pyroptosis in human neuroblast SH-SY5Y cells following MPP^+^ treatment, a common in vitro Parkinson’s disease cell model. Mechanistically, TXNIP ubiquitination through MIB1 E3 ubiquitin ligase regulates NLRP3 inflammasome activation in neuroblasts. In contrast, MPP^+^ activates the NLPR3 inflammasome through miR590-3p upregulation and direct interference with MIB1-dependent TXNIP ubiquitination. LncZFAS overexpression inhibits this entire pathway through direct interference with miR590-3p, exposing a novel research idea regarding the mechanism of Parkinson’s disease.

**Supplementary Information:**

The online version contains supplementary material available at 10.1007/s12035-021-02619-z.

## Introduction

Parkinson’s disease is one of the leading neurodegenerative diseases in developed countries, affecting 1–2% of the elderly population (above 65 years old)[1]. The World Health Organization predicts that the incidence of Parkinson’s disease will double by 2030[2], resulting in a significant loss of healthy life years. Parkinson’s disease syndrome is defined by tremor, rigidity, progressive akinesia, and/or postural disturbance. Parkinson’s disease is a multifactorial disease with both genetic and environmental factors, but the complete etiological scenario remains unknown. Nonetheless, Parkinson’s disease pathology is well defined and is primarily characterized by the loss of dopaminergic neurons in the substantia nigra[3]. Mechanistically, α-synuclein misfolding and aggregation appears to be central for disease progression, but mitochondrial dysfunction, dysfunctional protein clearance and ubiquitin/proteasome systems, and neuroinflammation have also been associated with Parkinson’s disease.

Without a well-defined etiological model, the development and identification of novel therapeutic targets must rely on high throughput screening in immortalized or primary neural cell lines[4]. SH-SY5Y human neuroblasts are commonly used as an alternative to laborious and highly heterogeneous primary dopaminergic neuron cultures from rat/mouse embryos[5, 6]. Regardless of the cell model, 1-methyl-4-phenylpyridinium (MPP^+^) is used to induce Parkinson’s disease-like cellular disease. MPP^+^ is a dopaminergic neurotoxin and the active metabolite of MPT (1-methyl-4-phenyl-1,2,3,6-tetrahydropyridine), which is known to cause human parkinsonism after injection[7].

Neuroinflammation was initially thought to be a side effect of Parkinson’s disease pathogenesis. However, this concept has now been revised, and microglial inflammatory signals are known to exacerbate and significantly contribute to Parkinson’s disease progression[8–11]. Inflammasomes sense microbial infection or host-derived danger signals indicative of metabolic perturbations[12]. These large multimeric caspase-1-activating complexes control the maturation and secretion of interleukins, such as IL-1b and IL-18, with potent proinflammatory activities against infection and injury[12, 13]. Many inflammasomes are activated through direct interaction with pathogen- or danger-associated molecular patterns (PAMPs and DAMPs, respectively)[12]. However, Nod-like receptor with pyrin domain (NLRP) 3 can sense intracellular metabolic perturbations, such as intracellular ATP and K^+^ ion imbalance or oxidative stress, through as yet unclear mechanisms[14]. Perhaps, for this reason, NLRP3 has been extensively studied in autoimmune disease and chronic inflammation[15, 16]. Furthermore, MPTP-driven NLRP3 inflammasome activation in microglia has recently been shown to play a central role in dopaminergic neurodegeneration and Parkinson’s disease[17]. NLRP3 is regulated by both post-transcriptional and post-translational signals[18]. In homeostasis, NLRP3 is generally expressed at extremely low levels, but is quickly and highly upregulated after PAMP or DAMP prime signaling[19]. In the cytosol, NLRP3 senses K^+^ efflux, intracellular oxidative stress, or extracellular elevated ATP levels[19]; any of these metabolic perturbations induce NLRP3 oligomerization, interaction with the adaptor protein ASC, and recruitment of cysteine protease pro-caspase-1[5]. Autocatalysis and activation of caspase-1 lead to cleavage, maturation, and secretion of proinflammatory cytokines, including IL-1β and IL-18, and, sometimes, induce programmed inflammatory cell death by pyroptosis[20–23]. NLRP3 activation promotes the secretion of the inflammatory cytokine interleukin-1β/18 (IL-1β/18) and induces pyroptosis to rupture microglia to further release inflammatory factor in Parkinson’s disease[24].

The next-generation sequencing revolution identified a plethora of long noncoding RNAs (lncRNAs), which were previously assumed to be biologically irrelevant, but are now known to have important transcriptional regulatory functions[25]. Recently, lncRNAs have also been shown to regulate protein expression post-transcriptionally through interference with microRNAs (miRs), smaller noncoding RNAs that directly bind to protein-coding mRNA, inhibiting translation and promoting transcript degradation[26, 27]. Moreover, a myriad of lncRNAs and miRs have been linked to the regulation of inflammatory signaling and neurodegenerative diseases, including IL-1β secretion and Parkinson’s disease[28, 29].

ZNFX1 antisense RNA 1 (ZFAS1) was recently identified as a novel lncRNA transcribed from the antisense orientation of zinc finger NFX1-type containing 1 (ZNFX1) located on chromosome 20q13.13[30]. Over the past 5 years, LncZFAS1 has emerged as a regulatory factor in multiple diseases, including acute myocardial infarction[31, 32], rheumatoid arthritis[33], and cancer[34]. Interestingly, much like Parkinson’s disease, myocardial infarction and rheumatoid arthritis are also driven and exacerbated by dysregulated inflammasome activation[35]. Furthermore, LncZFAS1 shows a broad molecular functional profile interfering in complex pathways including cell proliferation and cell death[36]. Therefore, we hypothesized that LncZFAS1 might regulate inflammasome activation and pyroptosis during Parkinson’s disease. Here, LncZFAS1 was identified as a regulator of inflammasome activation and pyroptosis in human neuroblast SH-SY5Y cells following MPP^+^ treatment. This work unveils a potential beneficial role of LncZFAS1 during Parkinson’s disease progression, which could be explored as a new research idea to elucidate the mechanism of Parkinson’s disease.

## Materials and Methods

### Tissue Culture

SH-SY5Y cells were acquired from ATCC and maintained in Dulbecco-modified Eagle medium (DMEM)/F12 (1:1) with penicillin (100 U/mL), streptomycin (100 μg/mL), and 10% fetal bovine serum (FBS). At 90% confluency, the monolayer was washed twice with phosphate buffered saline (PBS), and the cells were chemically detached with 0.05% trypsin for 2 min. After quenching with complete media, the cells were pelleted by centrifugation at 800 rpm for 5 min, washed twice with PBS, resuspended in complete medium and 3 × 10^4^ to 10^6^ cells seeded in 10-cm tissue culture dishes. The medium was changed every other day. SH-SY5Y cells were differentiated into dopaminergic neuron-like cells with (10 μM) retinoic acid in DMEM (1%FBS) for 5 days. On differentiation, cell adherence was confirmed under the microscope, and the remaining suspension cells were removed by gentle washing.

### Pyroptosis Assay

SH-SY5Y cells were seeded in 6-well plates at 10^6^ cells/well for 24 h and then treated with increasing MPP^+^ concentrations for 24 h. Cells were gently washed with PBS and chemically detached with 0.05% trypsin/EDTA. After detachment, trypsin was quenched with complete media, cells were pelleted by centrifugation, and the caspase activity was detected with the FAM-FLICA caspase-1 assay kit (ImmunoChemistry Technologies, LLC, Bloomington, MN, USA) according to the manufacturer’s recommendations. Briefly, cells were stained and incubated with FLICA (1:30) for 1 h at 37 °C protected from light. Cells were then by washed with 5 volumes of 1× wash buffer, pelleted by centrifugation, resuspended in 1× apoptosis wash buffer with 5 µL of propidium iodide (PI), and incubated for 10 min at 37 °C protected from light. Data were acquired with a Beckman DxFlex flow cytometer (Beckman, Brea, CA, USA) and analyzed with CytExpert (Beckman Coulter Inc, CA, USA). Single stain controls were used to calculate the “compensation,” which refers to the process of correcting fluorescence spillover, that is, removing the signal of any given fluorochrome from all detectors except the one devoted to measuring that dye. PI-positive cells were detected according to the operating procedure of PI-staining kit (KeyGEN Biotech, Nanjing, China)

### Total Protein Extraction

Cells were seeded in 6-well plates at 10^6^ cells/well and treated with 1 mM MPP^+^. When designated, cells were also treated with miRr590-3p mimic or inhibitor and corresponding negative controls. At designated timepoints, cells were washed with pre-cooled PBS and lysed with 1 mL of RIPA buffer supplemented with phenylmethyl sulfonyl fluoride (PMSF) for each 100 µL of sample. After full lysis, samples were centrifuged at 12,000 g, 4 ℃, for 5 min, the supernatant was immediately transferred to a clean pre-cooled tube and stored at −80 ℃ for later analysis. The total protein content was quantified by BCA method, in which 25 µg protein was added to 5×loading buffer and boiled for 10 min in a water bath for denaturing. Samples were stored at −20 ℃ until required for western blotting.

### Co-Immunoprecipitation (Co-IP) Assay

Protein extracts were prepared as described above. Protein A/G-agarose microspheres were washed twice with PBS and adjusted to a 50% agarose microsphere concentration in PBS. In detail, 100 μL of 50% of protein A/G-agarose microspheres was added to 1 mL of sample and incubated in a horizontal shaker at 4 °C for 10 min to remove unspecific binding. Samples were then centrifuged at 14,000 g for 15 min at 4 °C, and the supernatant was transferred to a clean centrifuge tube. Total protein was estimated by the BCA method and adjusted to 3 μg/μL with PBS. Immunoprecipitation microspheres were prepared in 500 μL with pre-titrated target antibody and 1 volume of bead mixed with 7 volumes of sample. Samples were incubated overnight at 4 ℃ with gentle shaking, followed by centrifugation at 14,000 g for 5 s. The precipitate was collected and washed three times with pre-cooled washing buffer (800 μL per wash). The pellet was resuspended in a suitable volume of loading buffer, and the supernatant was collected for further downstream SDS-PAGE western-blot analysis.

### Western Blotting

Denatured samples were loaded in 10% or 12% resolving and 5% stacking SDS-PAGE gels prepared in house. The samples were separated by electrophoresis in the MINI-PTET (BioRad, CA, USA) system at 120 V for 5 min (stacking) and 80 V (resolving) for approximately 30 min with pre-chilled 1× electrophoresis buffer. Five microliters of 3-color pre-stained protein ladder (Green, BioReseach LLC, LA, USA) was used as the standard for protein size estimation. For transfer, the PVDF membrane was pre-activated in methanol for 1 min and then immersed in the transfer buffer for 15 min. The samples were transferred with a semi-dry blot apparatus (BioRad, California, USA). The transfer efficiency was confirmed by Ponceau S staining. For specific protein expression, the membranes were incubated with primary antibody, at pre-titrated concentrations (Table 1) in Tris-buffered saline/0.05% Tween 20 (TBST) and self-sealing bags, followed by incubation overnight at 4 ℃. The membranes were then washed three times with TBST for 10 min with gentle rocking, and antibody binding was detected with appropriate HRP-conjugated secondary antibody at pre-titrated concentrations in ziplock bags for 1 h at room temperature. The membranes were washed a further three times with TBST. The membranes were developed with enhanced chemiluminescence (ECL) solution (Thermo Fisher Scientific, Pittsburgh, PA, USA) for 5 min, according to the manufacturer’s instructions. Membranes were imaged with Tanon 6600 Luminescent Imaging Workstation (Tanon, Shanghai, China), and the relative protein expression levels were quantified with Image Pro Plus 6.0 software (Media Cybernetics, Rockville, MD, USA). The expression level was calculated as [target protein gray value]/[internal reference protein gray value].

**Table 1:** Antibodies and dilution

Antibodies	Dilution (application)	Source and Cat#
NLRP3	1:1000 (WB)	Abcam-ab214185
Gsdmd	1:1000 (WB)	Abcam-ab210070
ASC	1:100 (IP)	Abcam-ab151700
TXNIP	1:1000 (WB), 1:200 (IP)	Abcam-ab188865
TRX1	1:100 (IP)	Abcam-ab26320
MIB1	1:1000 (WB)	Abcam-ab124929
IL1β	1:1000 (WB)	Abcam-ab229696
Ubiquitin	1:2000 (WB)	Abcam-ab7780
Caspase-1	1:1000 (WB)	CST-89332
GAPDH	1:10,000 (WB)	Abcam-ab181602

### RNA Extraction

SH-SY5Y cells were treated with MPP^+^ as described above. At 90% confluency or designated timepoints, the cells were chemically detached with trypsin and lysed with RNAiso plus Trizol at room temperature for 10 min, before the RNA was extracted according to the manufacturer’s recommendations. Briefly, 1/5 volume of chloroform was added, samples were shaken and let stand at room temperature for 5 min, followed by 15 min centrifugation at 12,000 g, 4 °C. The supernatants were transferred to a centrifuge tube, 1 volume of isopropanol was added, and the samples were inverted and mixed vigorously. After 10-min incubation on ice, the samples were centrifuged at 12,000 g, 4 °C, for 10 min, and the supernatant was discarded. The RNA was washed with 1 mL of 75% ethanol, air-dried (5–10 min), and resuspended in 20 µL of RNase-free water. The RNA content, purity, and quality were estimated with Nanodrop 2000 (Thermo Fisher, Pittsburgh, PA, USA) (2 µL of sample).

### MicroRNA Extraction

MiRNA was extracted with a tissue/cell miRNA extraction kit (Haigene, Harbin, China) according to the manufacturer’s recommendations. In brief, 300 μL of miRNA ReagentA was added, and the sample was mixed by inversion and incubated at room temperature for 5 min. After lysis, 250 μL of miRNA ReagentB was added, again mixed by inversion and centrifuged at 13,000 rpm for 5 min. The supernatant was transferred to a clean 1.5-mL tube, 200 μL of absolute ethanol was added and mixed vigorously, and the sample was incubated at room temperature for 5 min with shaking. After centrifugation at 13,000 rpm for 10 min, isopropanol was added (3:7 volume ratio), and the sample was inverted several times and loaded into the miRNA adsorption column. The column was washed twice with 75% absolute ethanol, dried for 10 min, and eluted with 30 μL RnaseFree TE buffer to a new tube by centrifugation at 13,000 rpm for 2 min.

### Quantitative Real-Time Polymerase Chain Reaction (qRT-PCR)

First-strand cDNA reverse transcription from mRNA was performed using the iScript cDNA Synthesis Kit (BioRad, Hercules, CA, USA) in 20-μL reactions with 2 μL of total RNA, according to the manufacturer’s recommendations. The reaction was performed at 25 °C for 5 min, 42 °C for 30 min, and 85 °C for 5 min, and cDNA was stored at −70 °C. qRT-PCR was performed using the Sofast EvaGreen Supermix system (BioRad, Hercules, CA, USA) in 20-μL reactions with a 1-μL cDNA template according to the manufacturer’s recommendations in an ABI 7500 real-time quantitative PCR instrument (American Applied Biosystems, USA). First-strand cDNA reverse transcription from miR and lncRNA was performed using the one-step miR RT kit (Takara, Japan) in 20-μL reactions with 4 μL of RNA template according to the manufacturer’s recommendations. The reaction was performed for 60 min at 37 °C, followed by 5 min at 95 °C. qRT-PCR was performed using the cDNA SYBR Green miRNA fluorescence quantitative PCR kit (Haigene, Harbin, China) in 20-μL reactions with 2.5 μL template cDNA. qRT-PCR was run in an ABI 7500 real-time quantitative PCR instrument (American Applied Biosystems, Foster, CA, USA). qRT-PCR protocols are shown in Table 2. Gene-target specific primers are shown in Table 3. Fold change differences in gene expression were calculated using the 2^−ΔΔ^Ct method.

**Table 2:** qRT-PCR protocol

Step1: 95 ℃	15 min
Step2: 95 ℃	5 s
55 ℃	5 s
70 ℃	30 s
Step2: 30–40 cycles	
Step3: 4 ℃	Dissociation analysis

**Table 3:** Primers used in quantitative real-time PCR

Gene target	Primers	Sequence 5′-3′
LncRNAZFAS1	Forward	5′-AACCAGGCTTTGATTGAACC-3′
	Reverse	5′-ATTCCATCGCCAGTTTCT-3′
TXNIP	Forward	5′-CAACTTGCTGCCCGACAAAA-3′
	Reverse	5′-TGGGTGGCATGCAAGGTATT-3′
MIB1	Forward	5′-TGGGGATTCATTGCTGCTAGAT-3′
	Reverse	5′-ACAGTGTAAGAGGGCTAGAGAC-3
GAPDH	Forward	5′-GGTCTCCTCTGACTTCAACA-3′
	Reverse	5′-GTGAGGGTCTCTCTCTTCCT-3′
Hsa-miR-590-3p	Forward	5′-AAAGATTCCAAGAAGCTAAGGGTG-3′
	Reverse	5′-CCTAACTGGTTTCCTGTGCCTA-3′
U6	Forward	5′-CTCGCTTCGGCAGCACA-3′
	Reverse	5′-AACGCTTCACGAATTTGCGT-3′

### Lentiviral Production

RNAi sequences were cloned into the GV493 plasmid and expanded in *Escherichia coli* DH5α cells in house (Fig. S1). The LncZFAS1 RNA sequence was cloned into pcDNA3.1 (Fig. S2). Lentiviral packaging was performed in 293 T cells seeded at 5 × 10^6^ cells/15 mL in T75 flasks in DMEM with 10% FBS for 24 h before transfection. At 70–80% confluency, cells were washed with PBS, 1 volume of serum-free medium was added, and the cells were incubated for 2 h at 37 °C 5% CO_2_. The transfection mixture was prepared with 20 μL of vector plasmid, mixed with 15 μL pHelper1.0 vector plasmid, 10 μL pHelper 2.0 vector plasmid, and GK transfection reagent (Genechem, Shanghai, China), in a 1 mL total reaction volume, and incubated at room temperature for 15 min. The transfection mixture was slowly added to the 293T cell culture (in serum-free medium) with gentle rocking and incubated at 37 °C, 5% CO_2_ for 6 h. The cells were gently washed with warm PBS, and 20 mL of complete medium (with 10% FBS) was added and incubated at 37 °C and 5% CO_2_ for 48 h. Supernatants were collected 48 h after transfection, centrifuged at 4000 g for 10 min at 4 °C to remove cell debris, and transferred to 40-mL ultracentrifuge tubes through a 0.45-μm filter. Lentiviral particles were pelleted by ultracentrifugation at 25,000 rpm, 4 °C, for 2 h, the media supernatant was discarded, and lentiviral particles were resuspended in the residual volume and transferred to a clean tube. Samples were centrifuged again at 10,000 rpm for 5 min, and the supernatant containing lentiviral particles was transferred to a clean tube. The lentivirus titer was detected in 293 T adherent cells. Cells were seeded in 96-well plates at 4 × 10^4^ cells/well, in a 100 μL medium and incubated for 24 h. Lentiviral preps were serial diluted (1/10) in serum free media (10 to 10^−10^), added to T293 cells, and incubated at 37 °C and 5% CO_2_ for 24 h. Following incubation, 100 μL of complete medium was added and the lentiviral titer measured after 4 days by fluorescence expression. The lentiviral titer was determined as transducing units (TU/mL) calculated as follows: $$TU/mL=\frac{\frac{P*N}{100}*V*1}{DF}$$.

### Lentivirus Transfection

SH-SY5Y cells were seeded in 24-well plates at 0.5 × 10^5^ cells/well and incubated at 37 °C, 5% CO_2_ overnight. Before transfection, cells were washed with PBS, and 500 μL of 0.8 μg/mL polybrene in a serum-free medium with 20 μL lentivirus at a pre-titrated MOI of 5 was added. Cells were incubated overnight at 37 °C and 5% CO_2_, the medium was removed and replaced with 1 mL of complete medium, and further incubated at 37 °C and 5% CO_2_. Transfected cells were expanded and subcultured at a 1:3 ratio. Forty-eight hours after subculture, cells were seeded in Petri dishes at 200 μg/mL puromycin; selection medium was changed every 3/4 days until clonal cell clusters appeared. Single cell clones were digested, transferred to 6-well plates, expanded, frozen, and stored in liquid nitrogen. Transfection efficiency was confirmed by western blot and qRT-PCR (Fig. S3).

### Luciferase Reporter Assay (pMIR-REPORT Fluorescent Reporter Gene)

The 3′UTR full length of the pre-selected target gene MIB1 was identified based on the TargetScan Human 7.2 website (http:www.targetscan.org/vert_72/). Primer 5 (Premier, California) was used to design primers (Table 3) with the 3′UTR fragment of the MIB1 gene containing the hsa-miR-590-3p binding site. The MIB1 sequence (with 3′UTR) was then cloned in house into the pmiR-report plasmid (Ambion, TX, USA, Fig. S4) and expanded in *E. coli* DH5α cells. First-strand cDNA reverse transcription from mRNA was performed using the iScript cDNA Synthesis Kit (BioRad, Hercules, CA, USA) as described above. The site directed mutagenesis was performed with a QuickMutagenesis Kit (Thermo Fisher Scientific, Pittsburgh, PA, USA) according to the manufacturer’s recommendations. SH-SY5Y cells at 50% confluency were then transfected in 96-well plates with Lipofectamine 3000 system (Thermo Fisher Scientific, Pittsburgh, PA, USA) according to the manufacturer’s recommendations. Luciferase double reporter gene expression was detected with the dual luciferase reporter gene detection kit (KeyGEN Biotech, Nanjing, China) according to the manufacturer’s recommendations. Briefly, after 36–48 h of plasmid co-transfection, the medium was discarded, and the cells washed with 100 μL 1× PBS. Next, 50 μL of 1× phosphate-buffered saline (PLB) was added to each well, and the plate was incubated for 20–30 min with shaking to ensure lysis. Subsequently, 10 μL of the supernatant was added to a 96-well white opaque microtiter plate (Thermo Fisher, Pittsburgh, PA, USA), followed by 100 μL of pre-mixed Luciferase Assay Reagent II. Plates were read in a dark chamber with a Berthold LB941 microplate multi-functional microplate reader (Berthold, Germany) 2 s after starting the reaction to detect the luciferase activity (RLU1). Finally, 100 μL of pre-mixed Stop&Glo Reagent was then added to each well to detect the intensity of the luciferase reaction in the internal reference control (RLU2). The gene-specific luciferase activity was calculated as RLU1/RLU2.

### miR590-3p Mimic and Inhibition Assay

The hsa-miR-590-3p mimics (sequence: UAAUUUAUGUAUAAGCUAGU), mimic NC (sequence: UUGUACUACACAAAAGUACUG), hsa-miR-590-3p inhibitor (sequence: ACUAGCUUAUACAUAAAAUUA), and inhibitor-NC (sequence: CAGUACUUUUGUGUAGUACAA) particles were purchased from Nanjing Darn Pharmaceutical Technology Co., Ltd (Nanjing, China). SH-SY5Y cells at 50% confluency were transfected into 6-well plates with Lipofectamine RNAiMAX (Thermo Fisher, Pittsburgh, PA, USA). In detail, Lipofectamine RNAiMAX (6 μL) and 20 pmol RNAi were mixed in 200 μL OPTI-Minimal Essential Medium (OPTI-MEM, Thermo Fisher Scientific, Pittsburgh, PA, USA) and incubated at room temperature for 20 min before transfection. SH-SY5Y cells were gently washed with 1 × PBS three times, 2 mL of OPTI-MEM medium was added to each well, and acclimated to 37 ℃ in 5% CO_2_. Transfection reagent mixture was added to the culture with gentle shaking, followed by incubation at 37 ℃, 5% CO_2_ for 48 h.

### Fluorescence In Situ Hybridization (FISH) and Confocal Microscopy

The FISH staining kit was purchased from RiboBio (Guangzhou, China). Digoxin labeled probes (TACTTCCAACACCCGCATTCATC) were acquired from RiboBio (Guangzhou, China). Briefly, 2 × 10^5^ SH-SY5Y cells were seeded onto 24-well microscopy slides (Thermo Fisher Scientific, Pittsburgh, PA, USA). After treatment, cells were washed with pre-cooled PBS and fixed with 4% RNase-free paraformaldehyde at room temperature for 15 min. After washing with PBS three times for 5 min, cells were permeabilized with 0.2–0.5% Triton X-100 for 5 min at room temperature. Cells were washed again with PBS (3 × 5 min) and dehydrated (80–90–100% alcohol gradient for 2–3 min each). The hybridization solution was prepared in house with formamide mixed with 2 × saline-sodium citrate (SSC) at a 1:1 volume at room temperature for 10 min. Cells were washed again with PBS, and 25 µL of hybridization solution was added to each well and incubated in the hybridization furnace at 50 ℃ for 4–8 h. Following incubation, 60 µL of denaturing hybridization solution containing probe was added to each well and incubated overnight in the hybridization furnace at 50 ℃. Following incubation, the cells were washed with 0.1 × SSC with 0.1% SDS and 50% formamide for 30 min. The samples were then blocked with 90 µL 20% sheep serum at room temperature for 1 h. Hybridization was detected with 60 µL anti-digoxin antibody, prepared 1/2500 in 10% sheep serum, and incubated overnight at 4 °C. After washing with PBS, samples were counterstained with 10 µL of 200 mg/mL DAPI at room temperature for 10 min. Cells were washed once with PBS, mounted with 10 µL anti-quenching mounting agent, sealed with rubber cement, and imaged using a Zeiss LSM laser confocal microscope (Zeiss, Germany).

### Statistical Analysis

All data are expressed as the mean ± SD. One-way ANOVA and non-parametric Kruskal–Wallis test were used to examine the statistical differences between groups. *p* values < 0.05 were considered to indicate significant differences. Data were analyzed with GraphPad Prism 6.0 (San Diego, CA, USA).

## Results

### MPP^+^ Induced Pyroptosis Through Inflammasome Activation in SH-SY5Y Neuronal Cells

**Fig. 1:**
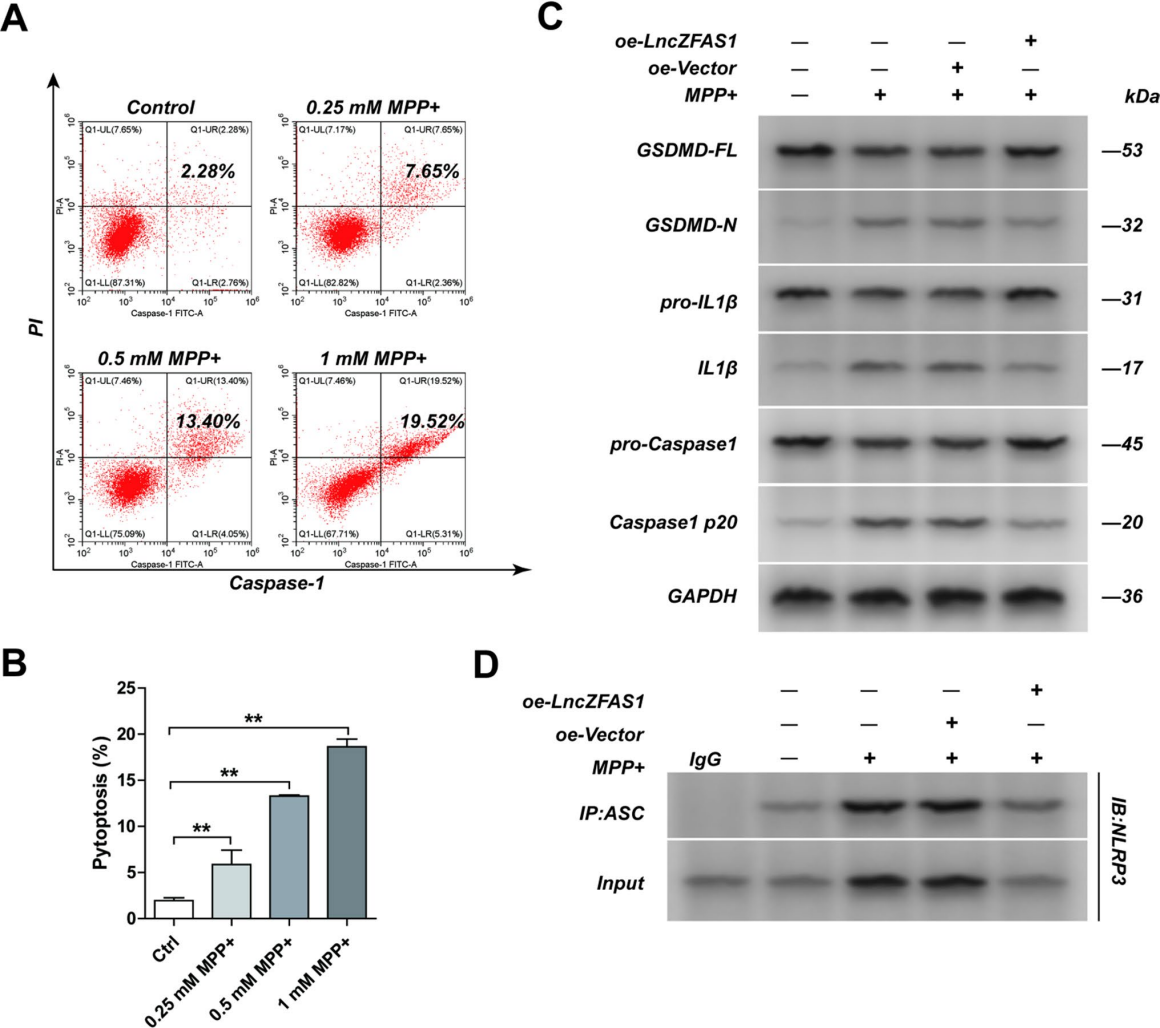
MPP^+^ induced SH-SY5Y cell pyroptosis by inflammasome activation. SH-SY5Y cells were treated with MPP^+^ (0.25, 0.5,1 mM) for 24 h and frequency of pyroptotic cells measure by flow cytometry. Frequency of pyroptotic cells was quantified by calculation of the internalization of PI and caspase-1 activation in the cells (**A**). Summary data for the frequency pyroptotic SH-SY5Y cells in the indicated groups as in **A**. The frequency of pyroptotic cells was increased along with the concentration of MPP^+^ (**B**). Expression of inflammasome effector proteins Gsdmd-full length, Gsdmd-N, pro-IL1β, IL-1β, pro-caspase-1, caspase-1 p20 in SH-SY5Y cells from the indicated group was detected by western blot assay. MPP^+^ treatment significantly induced cleavage of Gsdmd, pro-IL-1β, and caspase-1 and had no impact on the protein levels of Gsdmd, pro-IL-1β, and caspase-1 pre-cleaved peptides (**C**). NLRP3/ASC interaction detected following immunoprecipitation with anti-ASC or control IgG antibody (**D**). Results represent mean ± SD of three individual experiments for each condition, performed in triplicate. *p<0.05, **p<0.01

NLRP3 inflammasome activation and pyroptosis in primary microglial cells were found to be pivotal for MTPT-induced Parkinson’s disease progression in a murine model[17]. To determine whether MPP^+^ can also induce inflammasome activation and pyroptosis in human neuroblast cells, SH-SY5Y cells were treated with MPP^+^ in increasing concentrations, and the frequency of pyroptotic cells was quantified by PI internalization and caspase-1 activation. As expected, MPP^+^ treatment with increasing MTPT concentrations of 250 nM to 1 mM significantly increased the frequency of pyroptotic SH-SY5Y cells 24 h after treatment (Fig. 1A, B). Furthermore, MPP^+^ treatment significantly induced NLPR3/ASC association and cleavage of Gsdmd, pro-IL-1β, and caspase-1, all hallmarks of inflammasome activation. MPP^+^ had no impact on the protein levels of Gsdmd, pro-IL-1β, and caspase-1 pre-cleaved peptides (Fig. 1C, D).

### LncZFAS1 Inhibited MPP^+^-Induced Pyroptosis in SH-SY5Y Human Neuroblasts

**Fig. 2:**
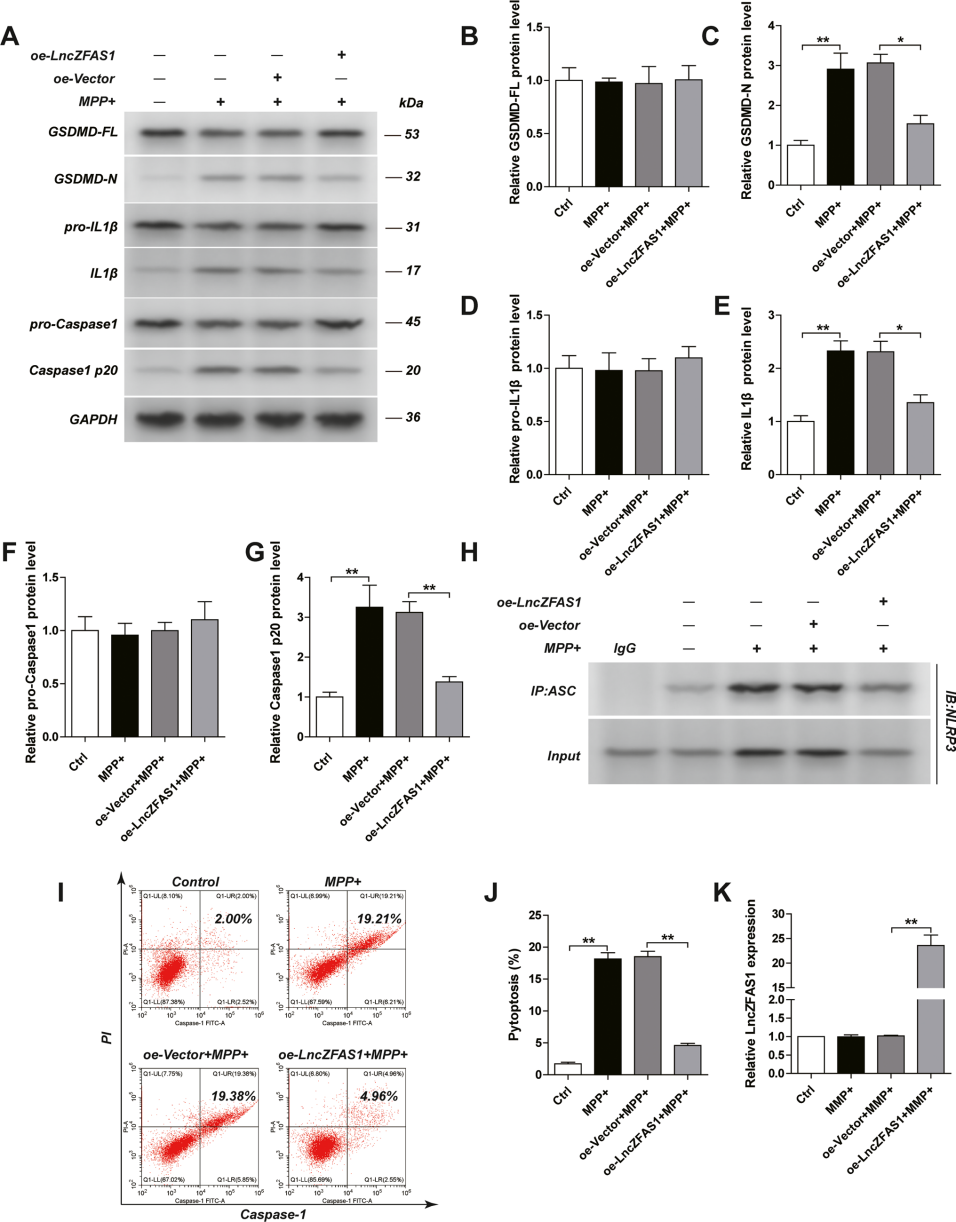
LncZFAS1 overexpression inhibited inflammasome activation and pyroptosis in SH-SY5Y cells. SH-SY5Y cells were stably transfected with lentiviral vector for LncZFAS overexpression or corresponding empty vector control. SH-SY5Y transfected cells were treated with MPP^+^ (1 mM) for 24 h and the expression of inflammasome effector proteins in SH-SY5Y cells detected by western blot assay (**A**). Relative expression of these proteins and their quantifications were shown respectively (**B**–**G**) (the same as followings). NLRP3/ASC interaction detected by immunoprecipitation with anti- ASC or control IgG antibody (**H**). Frequency of pyroptotic SH-SY5Y transfected cells following MPP^+^ treatment, measured by flow cytometry as described in Figure 1 (**I**,**J**). The expression of LncZFAS1 in the indicated group was measured by qPCR (**K**). Data represented as mean ± SD of three individual experiments for each condition, performed in triplicate. *p < 0.05, **p < 0.01

MicroRNA and LncRNA regulate a myriad of metabolic and inflammatory pathways, including inflammasome activation[25]. The role of LncZFAS in the inflammasome and pyroptosis has not yet been addressed. Therefore, to understand the impact of ZFAS in MPP^+^-induced pyroptosis, lncZFAS was stably transfected into SH-SY5Y cells before MMP^+^ treatment (Fig. S3). LncZFAS1 overexpressing (oe-LncZFAS1) cells showed significantly lower cleavage of Gsdmd, pro-IL-1β, and caspase-1 following MPP^+^ treatment compared to the empty vector transfected control cells (oe-vector), although there was no impact on the overall pre-cleaved peptide levels (Fig. 2A–G). Moreover, oe-LncZFAS1 cells had lower NRLP3/ASC association than oe-vector cells 24 h after MPP^+^ treatment (Fig. 2H). Consequently, oe-LncZFAS1 SH-SY5Y cells were resistant to MPP^+^-induced pyroptosis (Fig. 2I–K).

**Fig. 3:**
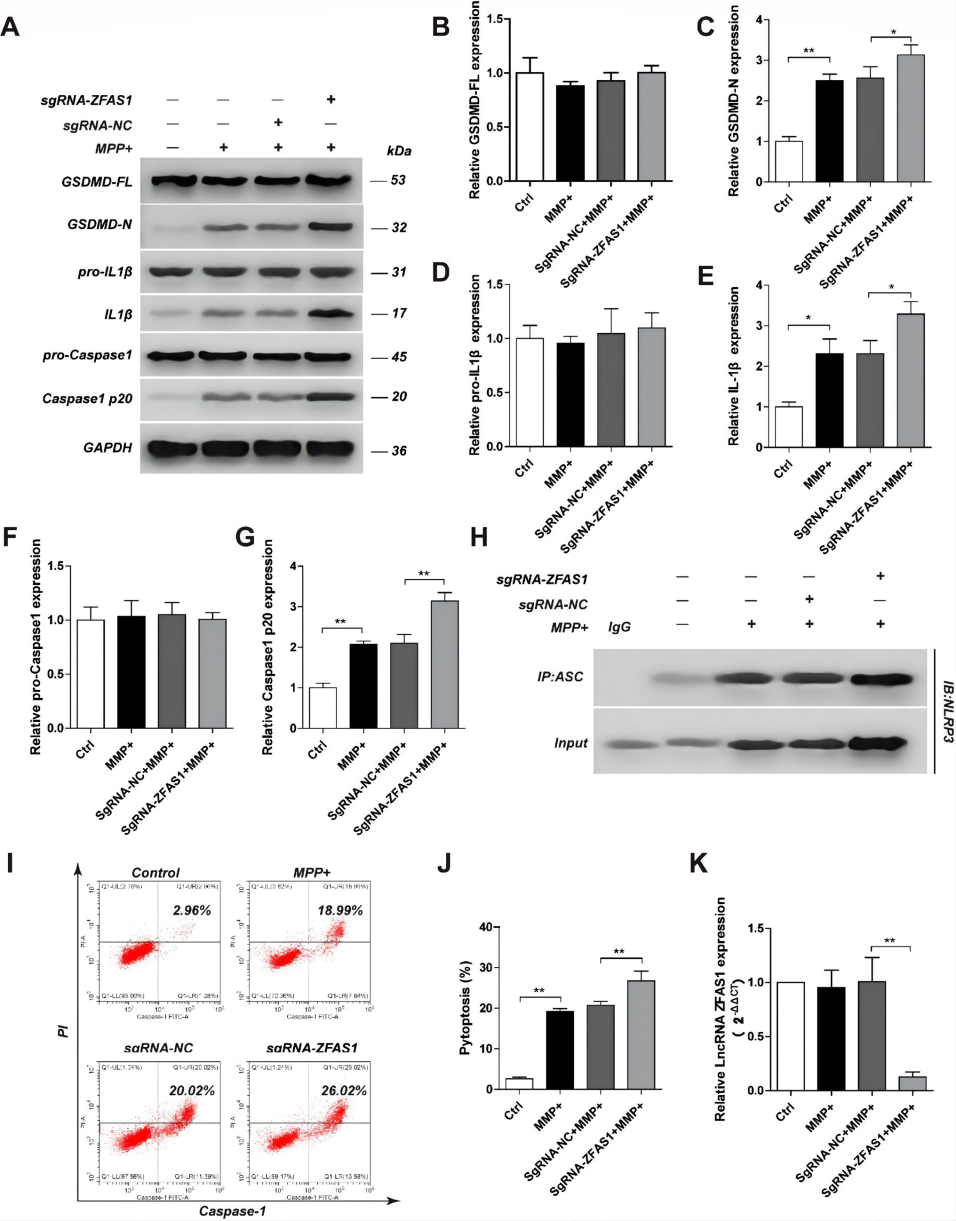
LncZFAS1 knockout induced inflammasome activation and pyroptosis in SH-SY5Y cells. LncZFAS1 was stably knockout by Crispr-cas9 plasmid in SH-SY5Y cells (sgRNA-ZFAS1) or corresponding negative control (sgRNA-NC), which were then treated with MPP^+^ (1 mM) for 24 h. Expression of inflammasome effector proteins of SH-SY5Y transfected cells was detected by western blot assay (**A**), and their quantifications was shown respectively (**B**–**G**). Cell lysates were immunoprecipitated with anti-ASC antibody or control IgG followed by Co-IP assay to determine the interaction of Nlrp3 and ASC (**H**). The pyroptosis ratio of SH-SY5Y cells in the indicated group was measured by flow cytometric analysis as above (**I**,**J**). The relative expression of LncZFAS1 was measured by qPCR (**K**). Data represented as mean ± SD of three individual experiments for each condition, performed in triplicate. *p < 0.05, **p < 0.01

To confirm the role of lncZFAS on MMP^+^-induced pyroptosis, lncZFAS was stably knocked-out in SH-SY5Y cells (sgRNA-ZFAS1) (Fig. 3). MPP^+^-treated sgRNA-ZFAS1 cells showed significantly higher cleavage of Gsdmd, pro-IL-1β, and caspase-1 than MPP^+^-treated control cells, although there was no impact on the overall pre-cleaved peptide levels (Fig. 3A–G). Additionally, sgRNA-ZFAS1 cells had higher NRLP3/ASC association than sgRNA-NC cells 24 h after MPP^+^ treatment (Fig. 3H). Finally, sgRNA-ZFAS1 cells showed higher sensitivity to MPP^+^-induced pyroptosis (Fig. 3I–K).

### LncZFAS1 Blocked MPP^+^-Induced Oxidative Stress Through the TRX1/TXNIP Redox Signaling Complex

**Fig. 4:**
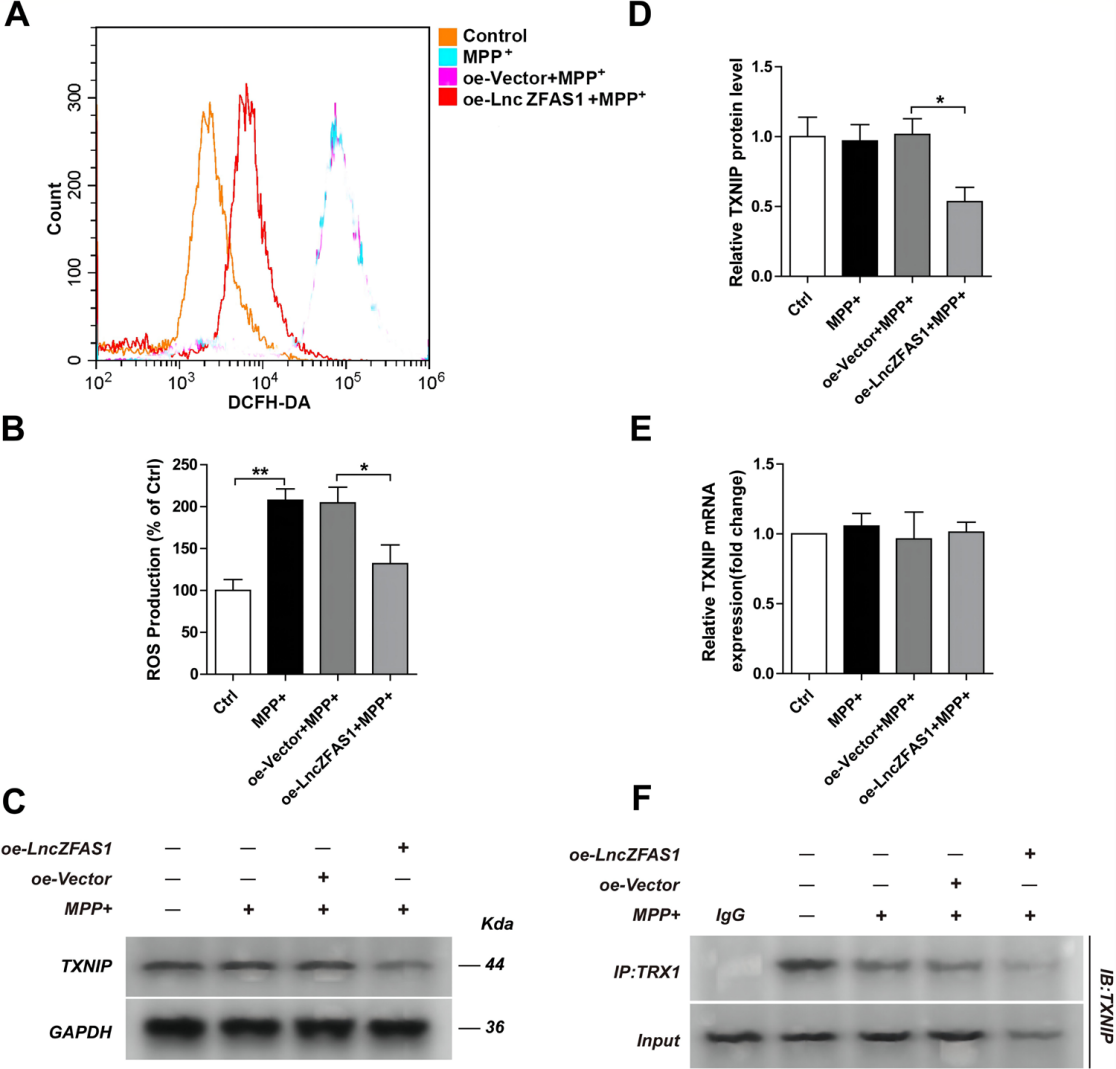
LncZFAS1 overexpression blocked MPP^+^-induced oxidative stress through TRX1/TXNIP redox signaling complex. SH-SY5Y cells were stably transfected with lentiviral vector for LncZFAS overexpression or corresponding empty vector control. SH-SY5Y transfected cells were treated with MPP^+^ (1 mM) for 24 h, and intracellular ROS production level was measured by flow cytometry (**A** and **B**). Protein and transcriptional expression of TXNIP was detected by western blot assay (**C**, **D**) or qRT-PCR (**E**). TXNIP/TRX1 interaction was detected by immunoprecipitation with anti-TRX1 antibody or control IgG (**F**). Data represented as mean ± SD of three individual experiments for each condition, performed in triplicate. *p < 0.05, **p < 0.01

Increased intracellular oxidative stress may act as second signal for NLRP3 inflammasome activation[18, 21–23]. To determine whether MPP^+^ induces intracellular oxidative stress in SH-SY5Y cells, intracellular ROS was measured using a DCFH-DA ROS-sensing fluorescent probe and analyzed by flow cytometry. As expected, MPP^+^ significantly induced intracellular ROS in SH-SY5Y human neuroblasts 24 h after treatment (Fig. 4A, B). Furthermore, to determine whether LncZFAS1 inhibits MPP^+^-induced oxidative stress, oe-SHSY5Y cells were treated with MPP^+^, and the intracellular ROS-levels were measured 24 h after treatment. Again, as hypothesized, LncZFAS1 overexpression significantly decreased intracellular oxidative stress compared to corresponding empty vector transfected control cells (Fig. 4A, B).

Increased oxidative stress activates the NLRP3 inflammasome through induction and activation of the TXNIP redox-sensing complex, but the defined molecular mechanism remains elusive[37, 38]. In SH-SY5Y cells, MPP^+^ did not change the TXNIP protein transcriptional and translational levels (Fig. 4C–E), nor did it change the TXNIP/TRX1 interaction (Fig. 4F). In contrast, LncZFAS1 significantly decreased post-translational TXNIP protein levels (Fig. 4C–E) with a prominent decrease in the TXNIP/TRX1 interaction (Fig. 4F).

**Fig. 5:**
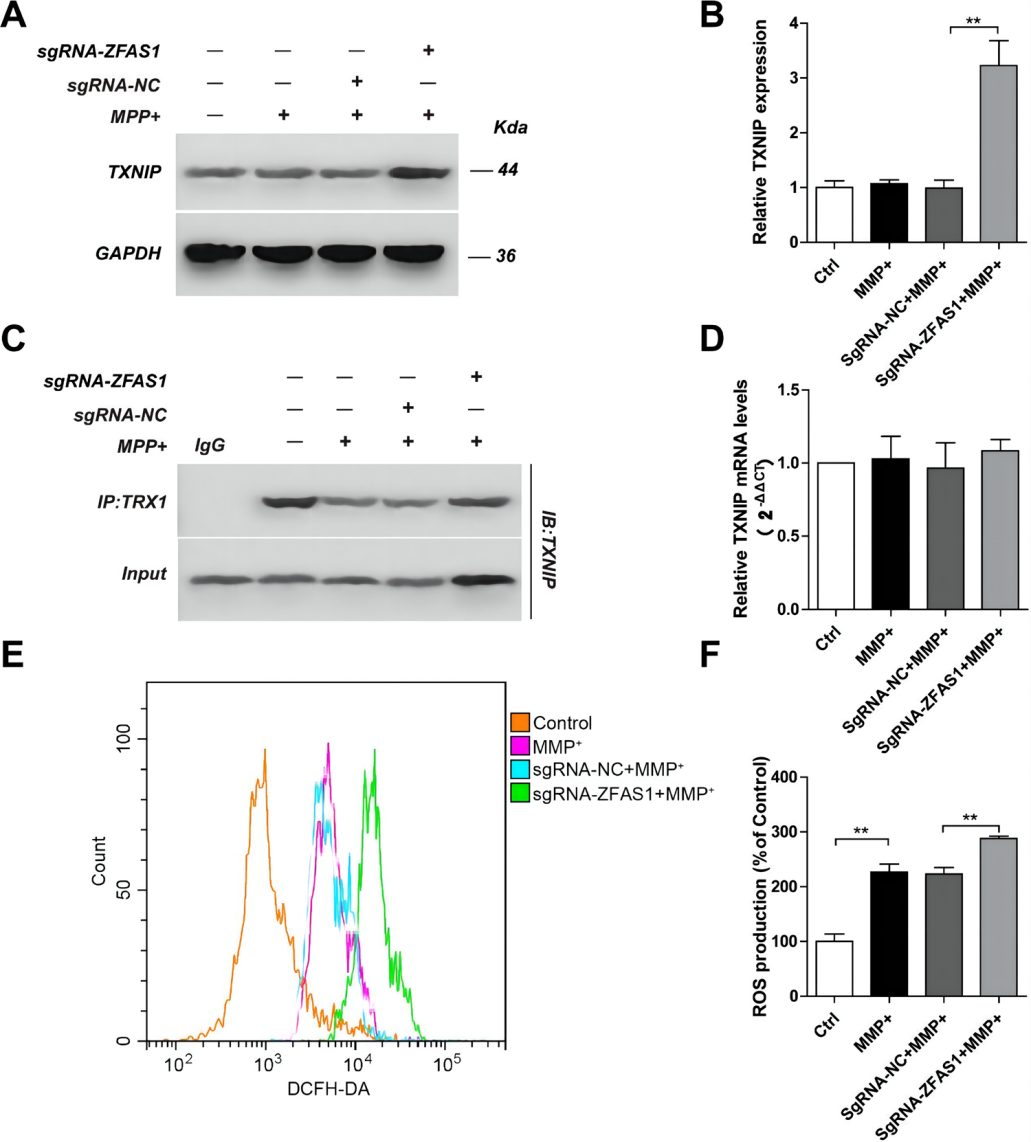
LncZFAS1 knockout enhances MPP^+^-induced oxidative stress through TRX1/TXNIP redox signaling complex. LncZFAS1 was stably knockout by Crispr-cas9 plasmid in SH-SY5Y cells (sgRNA-ZFAS1) or corresponding negative control (sgRNA-NC), which were then treated with MPP^+^ (1 mM) for 24 h. The expression of TXNIP (**A**, **B**) was detected by western blot assay, and the cell lysates were immunoprecipitated with anti-TRX1 antibody or control IgG followed by Co-IP assay to determine the interaction of TXNIP and TRX1 (**C**). TXNIP mRNA levels of the indicated groups were measured by qPCR (**D**). ROS production of sgRNA-ZFAS1 SH-SY5Y cells was measured by flow cytometric analysis (**E**–**F**). Data represented as mean ± SD of three individual experiments for each condition, performed in triplicate. *p < 0.05, **p < 0.01

To further validate the impact of the LncZFAS1 TXNIP redox-sensing complex in response to MPP^+^, the expression levels of TXNIP/TRX1 were measured in sgRNA-ZFAS1 cells. LncZFAS1 knockout significantly increased TXNIP protein levels but did not impact the transcriptional levels (Fig. 5A, B, and D). Moreover, after immunoprecipitation with an anti-TXNIP antibody, MPP^+^-treated sgRNA-ZFAS1 cells showed increased interaction with TRX1 compared to sgRNA-NC cells, likely due to increased TXNIP protein levels (Fig. 5C). Finally, to confirm that LncZFAS1 regulates the intracellular oxidative stress response to MPP^+^, ROS levels were measured in sgRNA-ZFAS1 cells and corresponding sgRNA-NC cells 24 h after MPP^+^ stimulation. In accordance with our previous observations, LncZFAS1 knockout significantly induced intracellular ROS production following MPP+ stimulation in SH-SY5Y cells (Fig. 5E, F).

### LncZFAS1 Inhibited Inflammasome Activation Through TXNIP Proteasomal Degradation

**Fig. 6:**
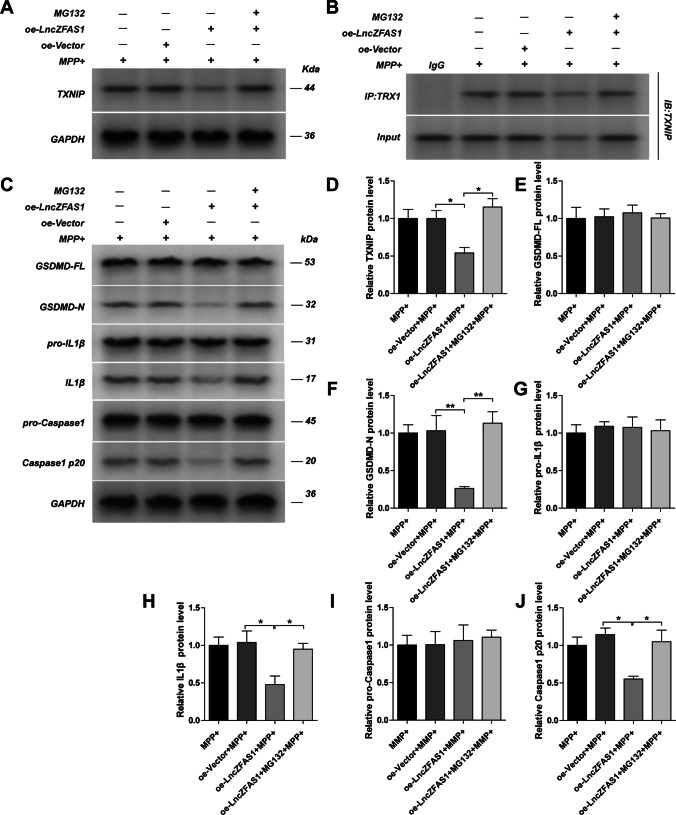
LncZFAS1 overexpression inhibited inflammasome activation through TXNIP proteasomal degradation. SH-SY5Y cells were stably transfected with lentiviral vector for LncZFAS overexpression or corresponding empty vector control. SH-SY5Y transfected cells were treated with MPP^+^ (1 mM) or co-treated with MG132 (1 μM) for 24 h and the expression of TXNIP (**A**) and inflammasome effector proteins were detected by western blot assay (**C**), and their quantifications were shown (**C**–**J**). TXNIP/TRX1 interaction was detected by immunoprecipitation with anti-TRX1 or control IgG antibody (**B**). Data represented as mean ± SD of three individual experiments for each condition, performed in triplicate. *p < 0.05, **p < 0.01

**Fig. 7:**
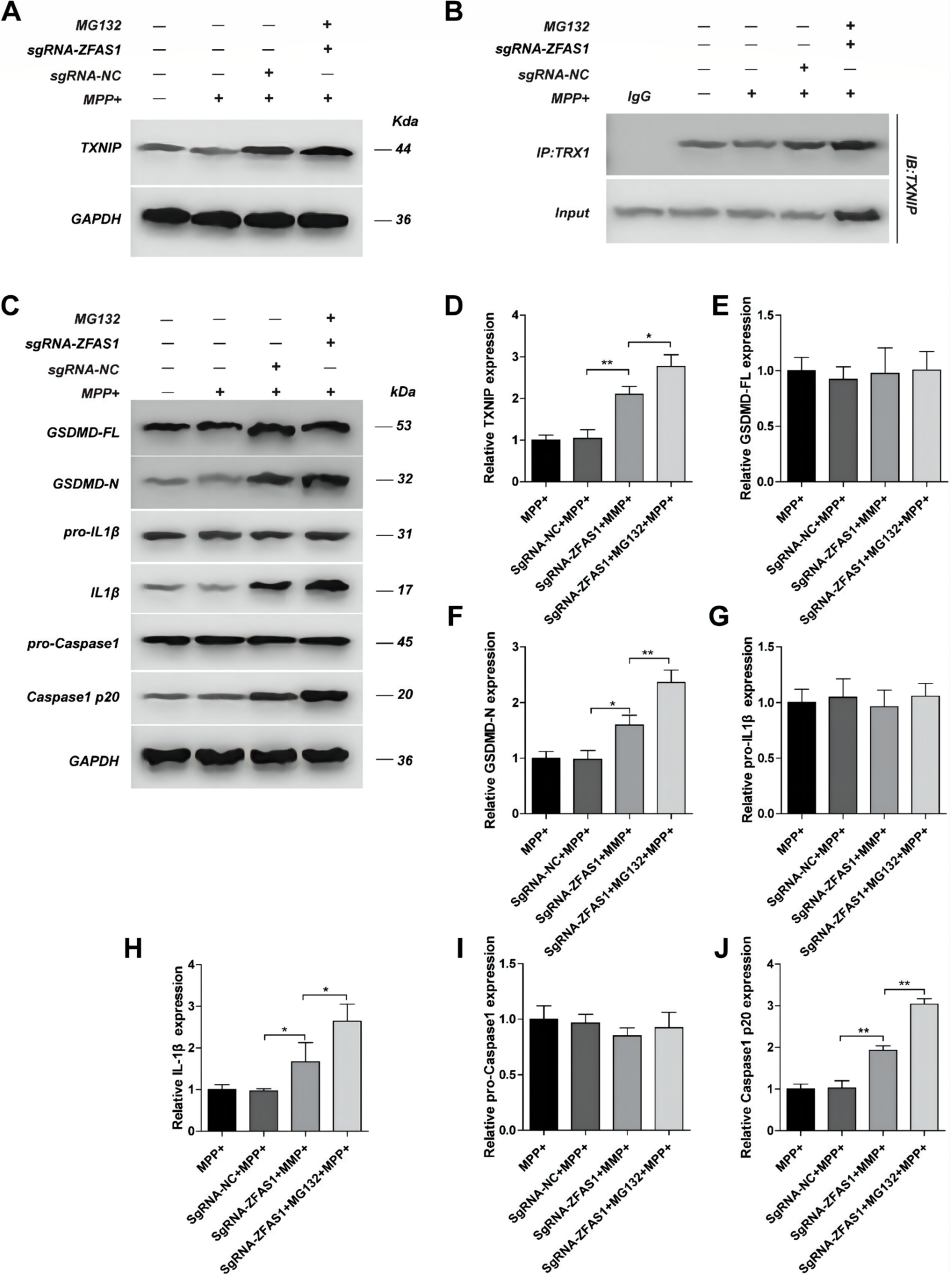
LncZFAS1 knockout increased TXNIP expression in MPP^+^-treated SH-SY5Y cells. LncZFAS1 was stably knockout by Crispr-cas9 plasmid in SH-SY5Y cells (sgRNA-ZFAS1) or corresponding negative control (sgRNA-NC), which were then treated with MPP^+^ (1 mM) or co-treated with MG132 (1 μM) for 24 h. The expression of TXNIP (**A**), inflammasome effector proteins of transfected cells were detected by western blot assay (**C**) and their responding quantification was shown respectively (**D**–**J**). TXNIP/TRX1 interaction was detected by immunoprecipitation with anti-TRX1 or control IgG antibody (**B**). Data represented as mean ± SD of three individual experiments for each condition, performed in triplicate. *p < 0.05, **p < 0.01

The proteasomal degradation pathway has been recently identified as a major regulator of inflammasome activation[39]. To determine whether LncZFAS1 post-translationally downregulates TXNIP through the proteasome degradation pathway, oe-ZFAS1 cells were treated with MPP^+^ and the MG132 proteasome inhibitor. As expected, proteasomal inhibition rescued TXNIP protein levels and TXNIP/TRX1 interactions in oe-ZFAS SY-SH5Y neuroblasts (Fig. 6A, B, D, and E). Consistent with the role of TXNIP in NLRP3 inflammasome activation, proteasomal inhibition rescued cleavage of Gsdmd, pro-IL-1β, and caspase-1 (Fig. 6C, F, H, and J), all of which are hallmarks of inflammasome activation in oe-ZFAS SY-SH5Y cells following MPP^+^ treatment. Proteasome inhibition had no impact on the protein levels of Gsdmd, pro-IL-1β, and caspase-1 pre-cleaved peptides (Fig. 6C, G, and I), while the opposite results were observed after LNCZFAS1 knockout (Fig. 7). Moreover, sgRNA-ZFAS1 cells showed increased TXNIP expression and TXR1 association even after treatment with the MG132 proteasome inhibitor (Fig. 7A, B), which resulted in increased inflammasome activation (Fig. 7C, D, F, H, and J). Nonetheless, LncZFAS1 knockout did not affect pre-cleaved levels of Gsdmd, pro-IL-1β, and caspase-1 (Fig. 7E, G, and I), again indicative of proteasomal regulation of the activated inflammasome.

### LncZFAS1 Induced TXNIP Proteosomal Degradation Through MIB1 Ubiquitination

**Fig. 8:**
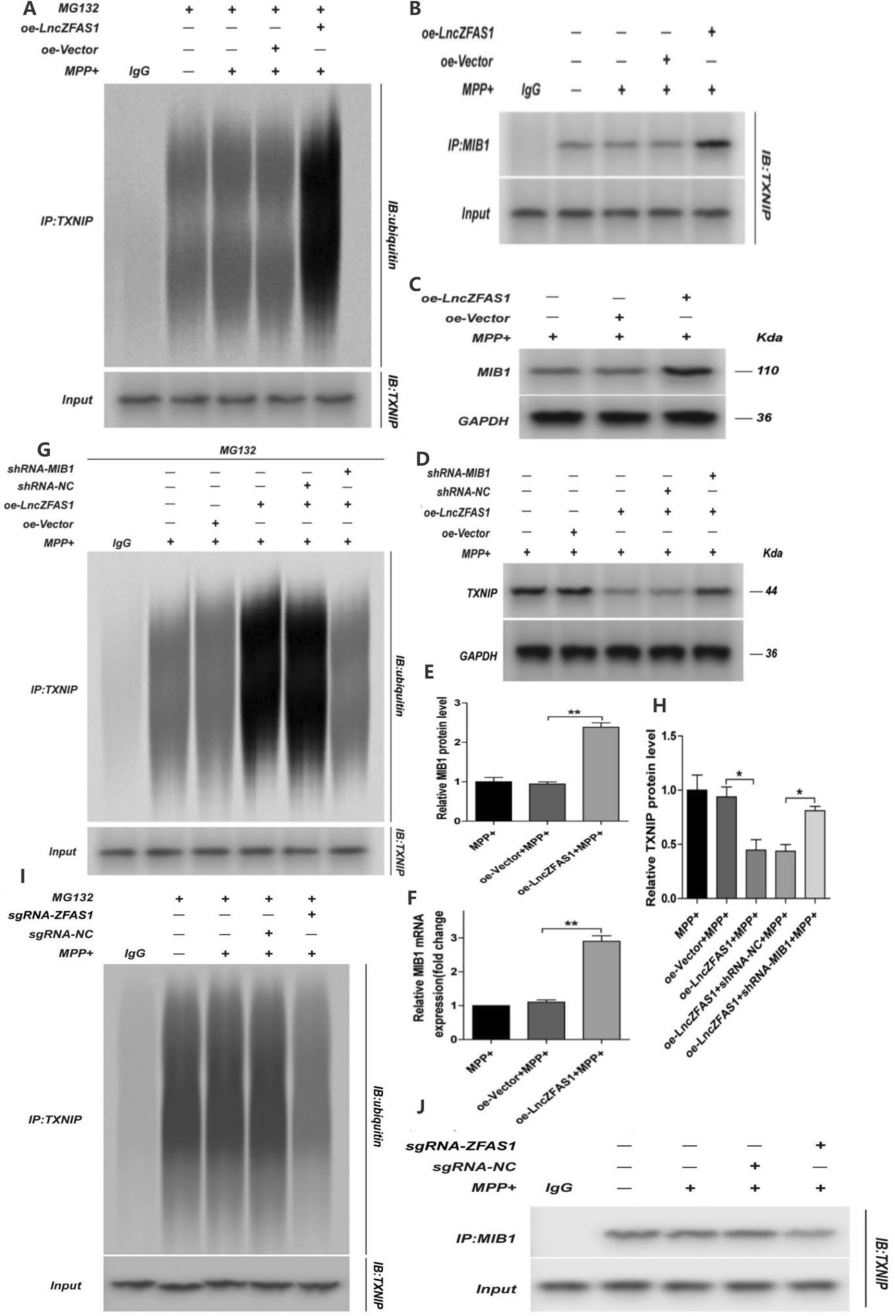
LncZFAS1 regulated TXNIP ubiquitination. SH-SY5Y cells were stably transfected with lentiviral vector for LncZFAS overexpression or corresponding empty vector control. Transfected cells were then treated with MPP^+^ (1 mM) or co-treated with MG132 (1 μM) for 24 h, and specific ubiquitination was assessed by immunoprecipitation with anti-TXNIP antibody, followed by immunoblot with anti-ubiquitin antibody (**A**). TXNIP/MIB1 interaction was determined by immunoprecipitation with anti-MIB1 or control IgG antibody (**B**). MIB1 expression was quantified by western blot (**C**, **E**) and qRT-PCR (**F**). A, B, C, G, and H results were representative of three independent experiments. LncZFAS1 stably knockout SH-SY5Y cells (sgRNA-ZFAS1) were treated with MPP^+^ (1 mM) or co-treated with MG132 (1 μM) for 24 h and cell extracts immunoprecipitated with anti-TXNIP antibody, followed by immunoblot with anti-ubiquitin antibody (**I**). TXNIP/MIB1 interaction was detected by immunoprecipitation with anti-MIB1 antibody or control IgG (**J**). Data represented as mean ± SD of three individual experiments for each condition, performed in triplicate. *p < 0.05, **p < 0.01

Proteasomal degradation is mainly regulated by the ubiquitin system[39]. Hence, the levels of TXNIP ubiquitination in oe-LncZFAS1 SH-SY5Y cells were assessed by Co-IP and western blotting (Fig. 8A). oe-LncZFAS1 cells showed higher TXNIP association with E3 ubiquitin ligase protein MIB1 following MPP^+^ treatment (Fig. 8B). Moreover, LncZFAS1 overexpression significantly induced MIB1 transcriptional and protein levels in SH-SY5Y cells following MPP^+^ treatment (Fig. 8C, E, and F). To verify that LncZFAS1-induced TXNIP ubiquitination depends on the MIB1 protein interaction, a MIB1 knockdown SH-SY5Y cell line was generated on the oe-ZFAS1 background (Fig. S3). MIB1 knockdown was shown to rescue TXNIP protein levels in oe-ZFAS1 cells and reduced TXNIP ubiquitination following MPP^+^ treatment (Fig. 8D, G, and H). In contrast, transfection with lentiviral scramble control had no impact on TXNIP protein levels or ubiquitination (Fig. 8G–H). A series of similar experiments was conducted with sgRNA-ZFAS1 cells, showing decreased TXNIP ubiquitination and MIB1 association (Fig. 8I, J).

### LncZFAS1 Interferes with miR590-3p-Mediated MIB-1 Downregulation

**Fig. 9:**
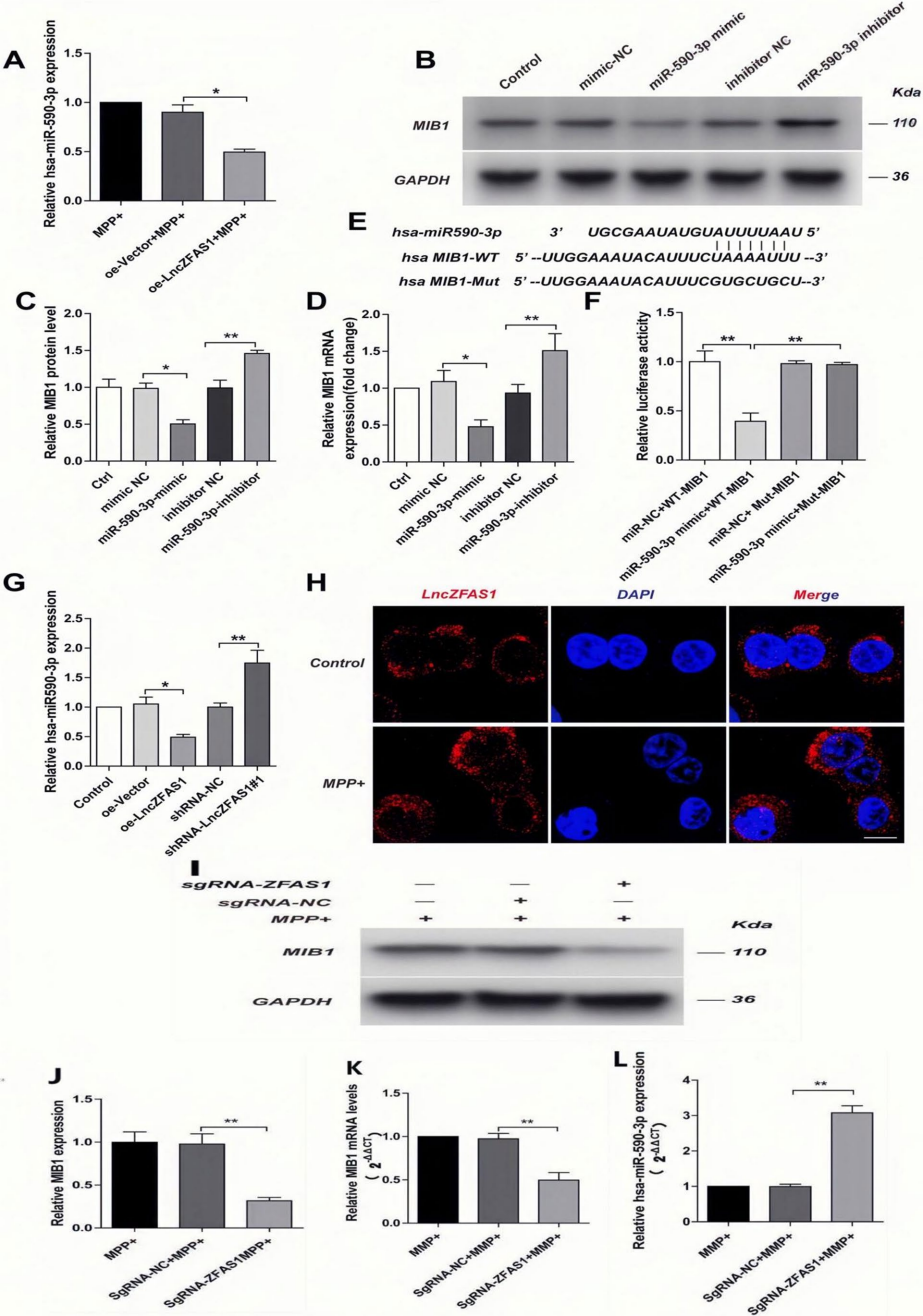
LncZFAS1 regulated miR590-3p-mediated MIB-1 inhibition. SH-SY5Y cells were stably transfected with lentiviral vector for LncZFAS1 overexpression or corresponding empty vector control, treated with MPP^+^ (1 mM) for 24 h, and Mir590-3p transcriptional levels were measured by qRT-PCR (**A**). Sh-SY5Y cells were treated with miR590-3p mimic, miR590-inhibitor, or corresponding controls, and MIB1 levels measured by western blot (**B**, **C**) or qRT-PCR (**D**). A MIB1 luciferase reporter system was generated and the miR-590-3p 3′UTR target sequence mutated for resistance (**E**). Transfected cells were treated with miR590-3p mimic, miR590-inhibitor, or corresponding controls and MIB1 levels measured by luciferase activity (**F**). SH-SY5Y cells were stably transfected with lentiviral vector for LncZFAS1 overexpression, ShRNA-ZFAS1 for LcnZFAS1 knockdown, or corresponding controls, and miR509-3p levels measured by qRT-PCR (**G**). SH-SY5Y cells were treated with MPP^+^ (1 mM) for 24 h and LncZFAS1 intracellular localization determined by FISH staining and confocal microscopy. LncZFAS1 stably knockout SH-SY5Y cells (sgRNA-ZFAS1) were treated with MPP^+^ (1 mM) for 24 h and MIB1 expression detected by western blot assay (**I**, **J**). MIB1 (**K**) and has-miR-590-3p (**L**) transcriptional levels were measured by qPCR. **A**, **B**, and **H** results were representative of three independent experiments. **C**, **D**, **F**, **G**, **J**, **K**, and **L** data represented as mean ± SD of three individual experiments for each condition, performed in triplicate. *p < 0.05, **p < 0.01

miRs are single small non-coding RNAs that regulate gene expression of a myriad of protein targets[40]. LncZFAS1 significantly downregulates miR590-3p, a putative regulator of MIB1 (Fig. 9A). To determine whether miR590-3p regulates MIB1 expression, SH-SY5Y cells were treated with a miR590-3p or a miR590-3p inhibitor and respective negative controls, and MIB1 protein levels were assessed by western blot. Consistent with the in silico predictions, miR590-3p treatment prominently decreased MIB1 protein expression. In contrast, the miR590-3p inhibitor greatly increased MIB1 protein levels (Fig. 9A, B). MiR regulated protein expression post-transcriptionally through direct binding to the 3′-untranslation region (3′UTR), inducing translation inhibition or mRNA degradation of their targets. Consistent with this mechanism, SH-SY5Y cells treated with miR590-3p had significantly lower MIB1 mRNA transcripts, while miR590-3p inhibition significantly increased MIB1 transcriptional expression (Fig. 9C). To validate direct miR590-3p binding to the 3′UTR, a MIB1 luciferase reporter system was generated with the WT MIB1 3′UTR (WT-MIB1) or a miR590-3p resistant 3′UTR (mut-MIB1) (Fig. 9D). Consistent with the transcriptional data, miR590-3p significantly decreased MIB1-luciferase activity in WT-MIB1 cells but had no impact on mut-MIB1 (Fig. 9E).

LncZFAS1 also regulates the activity of other miRs[41]. To determine whether LncZFAS1 upregulates MIB1 through interference with miR590-3p, the transcriptional levels of miR590-3p were quantified in oe-LnccnZFAS1 cells. The results showed that oe-LnccnZFAS1 SH-SY5Ycells had significantly lower miR590-3p transcripts than the corresponding oe-vector control cells. In contrast, LncZFAS1 knockdown significantly increased miR590-3p expression (Fig. 9F). Finally, to confirm that LncZFAS1 regulates miR590-3p pro-transcriptionally, the intracellular localization of LncZFAS1 was assessed by FISH staining and confocal microscopy. Consistent with the post-transcriptional regulation mechanism, LncZFAS1 expression was found to be mainly localized in the SH-SY5Y cell cytoplasm following MPP^+^ treatment (Fig. 9G). In contrast, sgRNA-ZFAS1 cells showed decreased MIB1 transcriptional and protein levels (Fig. 9I–K) and increased miR590-3p expression (Fig. 9L).

## Discussion

**Fig. 10:**
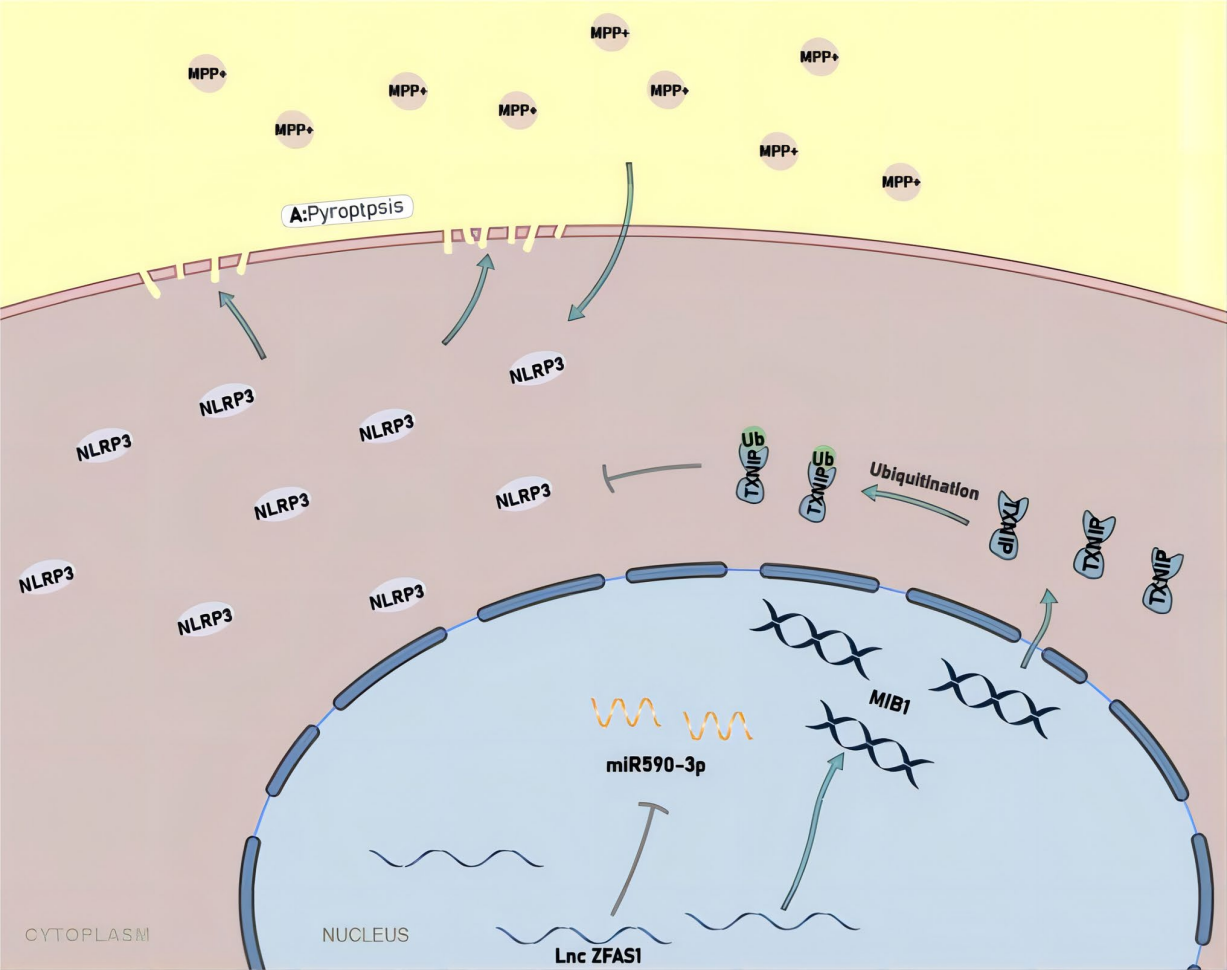
Molecular mechanism for LncZFAS1 mediated inflammasome regulation in neuronal cells. Under homeostasis MIB1-mediated TXNIP ubiquitination inhibited inflammasome activation to maintain a tolerogenic environment. Upon a neuroinflammatory signal (MPP^+^) miR590-3p upregulation inhibits MIB1 ubiquitin ligase, decreasing cytoplasmic Ub-TXNIP. In parallel, increased intracellular ROS activated the Txnip/TRX redox-sensing complex driving NLRP3/ASC association, leading to NLRP3 inflammasome activation and break of tolerance. lncZFAS upregulation inhibited this entire pathway through direct interference with miR590-3p, preventing MIB1 ubiquitin ligase inhibition

Exploration of the non-coding genome unveiled a panoply of formerly unknown lncRNAs with critical regulatory functions in the pathophysiology of many neurological diseases[42–48]. LncRNAs are mainly expressed during cellular senescence, which represents a major risk factor during neurodegenerative disease development[46, 49]. Moreover, recent literature has shown that lncRNAs have important roles in regulating the expression of nearby protein-coding genes, and that deregulation of this relationship may lead to brain diseases. Most lncRNAs expressed in the nervous system have only been identified in genome-wide expression screens, but their involvement in Parkinson’s disease is now a prolific research field[50–53]. This study showed that LncZFAS1 significantly regulated MPP^+^-induced pyroptosis and inflammasome activation in human neuroblasts. To our knowledge, this is the first report of a lncRNA directly regulating inflammasome activity in human neuroblasts, exposing lncZFAS as novel potential therapeutic approach for Parkinson’s disease (Fig. 10).

Clinical and experimental research suggests that microglial activation and neuroinflammation are key regulators of dopaminergic neuronal loss in Parkinson’s disease[54, 55]. Chronic activation of microglia and an excessive proinflammatory milieu in the brain can result in the expression of costimulatory molecules, neuroinflammation, and neuronal dysfunction[56–59]. Microglial-mediated neuroinflammation has been reported in many neurodegenerative disorders, and uncontrolled NLRP3 inflammasome activation in microglial cells has been observed in the tissue of the substantia nigra in the midbrain of patients with Parkinson’s disease[56, 60]. Besides, NLRP3 inflammasome activation may aggravate dopaminergic neuronal loss in Parkinson’s disease[24]. Both in vitro and in vivo models of Parkinson’s disease have suggested a link between the aggregation of α-synuclein, increased mitochondrial ROS, and cathepsin B release with the activation of microglial NLRP3 inflammation-mediated pyroptotic cell death of dopaminergic neurons in the substantia nigra[61–63]. In contrast, rare mutations in the NLRP3 inflammasome have been associated with a decreased risk of Parkinson’s disease[64]. Furthermore, several etiological factors associated with neuroinflammation and dopaminergic neuronal loss, such as mitochondrial generation of ROS, mitophagy, loss of function of dopaminergic receptors, and lncRNA, are frequently connected with microglial NLRP3 inflammasome activation[63, 64]. Effectors of pyroptosis are activated and transferred to the membrane to induce glial rupture, releasing more inflammatory mediators, which promote the progression of Parkinson’s disease with NLRP3[24]. Thus, the NLRP3 pathway may provide a new therapeutic avenue for Parkinson’s disease treatment. However, the exact mechanism underlying NLRP3 activation in neural cells remains unclear, and a deeper understanding is necessary before this pathway can be targeted efficiently. Here, MPP^+^ treatment significantly induced NLRP3 activation and pyroptosis in neuroblasts. Moreover, NLPR3 activation, ASC recruitment, caspase-1 cleavage, and IL-1β maturation were found to depend on the TXNIP/TRX1 interaction.

The thioredoxin (Trx) system (composed of NADPH, thioredoxin reductase, and Trx) is a key antioxidant system that protects cells from oxidative stress. Trx1 is a 12-kDa ubiquitous protein with disulfide-reducing activity, which is mainly localized in the cytoplasm[65]. TXNIP acts as an endogenous Trx inhibitor[66], and together, the Trx-Txnip complex has been recently described as novel protein signaling pathway for transducing redox-related signals[65]. TXNIP has a specific arrestin-like domain, which is responsible for highly promiscuous protein–protein interactions. Until now, TXNIP has been identified in association with importin-α, transcriptional co-repressors SMRT-mSin3-HDAC (histone deacetylase), Jab1, E3 ubiquitin ligase ITCH, Mybbp1a, and NLRP3, as well as Trx [37, 67–70]. Additionally, TXNIP-associated NLRP3 inflammasome activation plays an important role in degenerative and ischemic diseases such as Alzheimer’s disease, Parkinson’s disease, and stroke[71–73]. It has been observed that the expression of TXNIP was increased and knockdown of TXNIP by siRNA attenuated the NLRP3 inflammasome activation response in αSynagg-stimulated mouse microglial cells[73]. Together, these findings revealed a central role of TXNIP in a redox signaling complex that results in inflammasome activation. In agreement with this hypothesis, decreased TXNIP expression in neuroblasts significantly decreased MPP^+^ inflammasome activation. A TXNIP centered inflammasome regulation mechanism has not yet been reported. Here, we showed that increased TXNIP ubiquitination, through MIB1 E3 ubiquitin ligase, regulates NLRP3 inflammasome activation in neuroblasts. Mechanistically, MPP^+^ activated the NLPR3 inflammasome through miR590-3p upregulation, which in turn inhibited MIB1-dependent TXNIP ubiquitination. The increase in intracellular ROS then activated the TXNIP/TRX redox-sensing complex driving NLRP3/ASC association, caspase 1 activation, cytokine maturation, Grdm cleavage, and pyroptosis. In contrast, lncZFAS could inhibit this entire pathway through direct interference with miR590-3p, exposing it as a new research avenue in exploring the mechanism of inflammasome activation and pyroptosis in neuroblasts and Parkinson’s disease.

In this study, the Parkinson’s disease-associated neuroinflammatory response was investigated using MPTP-induced cells (MPP^+^ SH-SY5Y cells), which, compared to microglia, are rarely used in this field. The results showed that MPTP-induced NLRP3 inflammasome activation in microglia played a central role in dopaminergic neurodegeneration and Parkinson’s disease[17, 74]. NLRP3 promoted the secretion of IL-1β/18 and pyroptosis to rupture microglia to further release inflammatory factors in Parkinson’s disease[24, 75, 76]. The changes in NLRP3 were observed in the MPTP-induced SH-SY5Y cell lines. Krishna et al. found that context-specific network integrity ranked ROS metabolism as the most intact, followed by the ubiquitin proteasome system, dopamine metabolism, calcium signaling, mitochondria, and glycolysis in different Parkinson’s disease-related sub-systems of undifferentiated SH-SY5Y cells[77]. Thus, somatic mutations did not significantly alter Parkinson’s disease-related pathways in the undifferentiated SH-SY5Y cells. Furthermore, MPP^+^ inhibits complex I of the electron transport chain, increasing reactive ROS production, and redistributing DA to the cytosol to oxidize DA and generate more ROS[78]. Thus, SH-SY5Y cells are an appropriate model to study the effects of Parkinson’s disease-related mitochondrial inhibition on cell viability and function[79, 80]. Mitochondrial reaction and ROS metabolism are important steps in the chain of the neuroinflammatory response. Therefore, MPTP-induced SH-SY5Y may be used as an alternative to study the Parkinson’s disease neuroinflammatory response, which will extend the application of SH-SY5Y cell lines.

This study has several limitations. First, the characterization of the SH-SY5Y cell line showed moderate activity of dopamine-β-hydroxylase and negligible levels of choline acetyl-transferase and acetylcholinesterase, and butyryl-cholinesterase, basal noradrenaline release, and tyrosine hydroxylase activity. Therefore, the SH-SY5Y cell line may display a catecholaminergic and not purely dopaminergic phenotype[79]. Second, the SH-SY5Y cell line was obtained as a neuroblastoma derivative, with cancerous properties that may have influenced the differentiation fate, viability, growth performance, metabolic properties, and genomic stability. Hence, SH-SY5Y cells possess physiological characteristics that differ greatly from the normal dopaminergic neuronal features[79]. Finally, although most of the genes belonging to the major Parkinson’s disease pathways and modules were intact in the undifferentiated SH-SY5Y genome[78], it was unclear how the genes and modules were expressed in the MPP^+^ SH-SY5Y cell line and what the differences in gene expression were between human Parkinson’s disease and the MPP^+^ SH-SY5Y cell line. Thus, the MPP^+^ model was not completely representative of neuroinflammation and pyroptosis pathology in human Parkinson’s disease; however, this model still represents a direction worth exploring to maximize the benefit from SH-SY5Y cell line. Parkinson’s disease-related neuroinflammation or pyroptosis should be explored in further studies using primary dopaminergic neuron-like cells differentiated from patient-derived induced pluripotent stem cells, other cellular neurotoxin models, genetic models, microglia, and animal models, including mouse, rat, and fruit fly.

In conclusion, lncZFAS overexpression inhibited the TXNIP/MIB1 E3 ubiquitin ligase/NLRP3 pathway through direct interference with miR590-3p, which shows a novel research avenue in exploring the mechanism of inflammasome activation and pyroptosis in Parkinson’s disease.

## Supplementary Information

Below is the link to the electronic supplementary material.

Supplementary Fig. 1 GV493 vector used for lncZFAS1 cloning and lentiviral production (PNG 4682 KB)

High Resolution Image (TIF 370 KB)

Supplementary Fig. 2 pcDNA3.1-GFP vector used for lncZFAS1 overexpression in SH-SY5 cells (PNG 18054 KB)

High Resolution Image (TIF 1337 KB)

Supplementary Fig. 3 LncRNA ZFAS transcriptional levels in empty vector control and LncRNA ZFAS transfected SH-SY5Y cells, measured by qRT-PCR (PNG 5337 KB)

High Resolution Image (TIF 500 KB)

Supplementary Fig. 4 MIB1 sequence cloned in house into the pmiR-report plasmid (PNG 20740 KB)

High Resolution Image (TIF 4570 KB)

## Data Availability

Not applicable.
