# Supplementary figures and images for: A Novel Long-Noncoding RNA LncZFAS1 Prevents MPP+-Induced Neuroinflammation Through MIB1 Activation
Source: Mol Neurobiol. 2021 Nov 13;59(2):778–99. doi: 10.1007/s12035-021-02619-z (PMC8857135; doi:10.1007/s12035-021-02619-z)

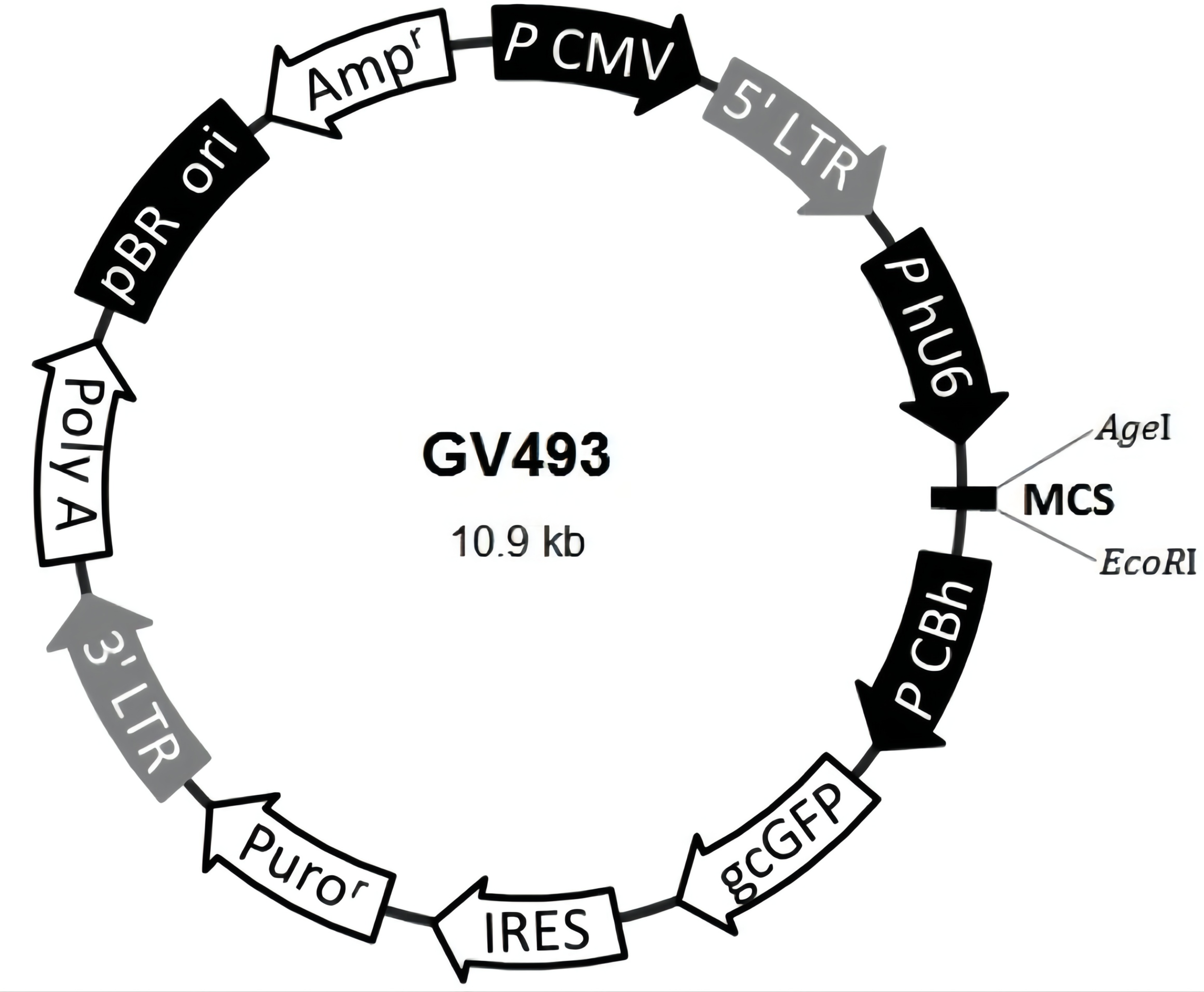

Supplement: Supplementary file 1 — GV493 vector used for lncZFAS1 cloning and lentiviral production (PNG 4682 KB) [file 12035_2021_2619_Fig11_ESM.png]

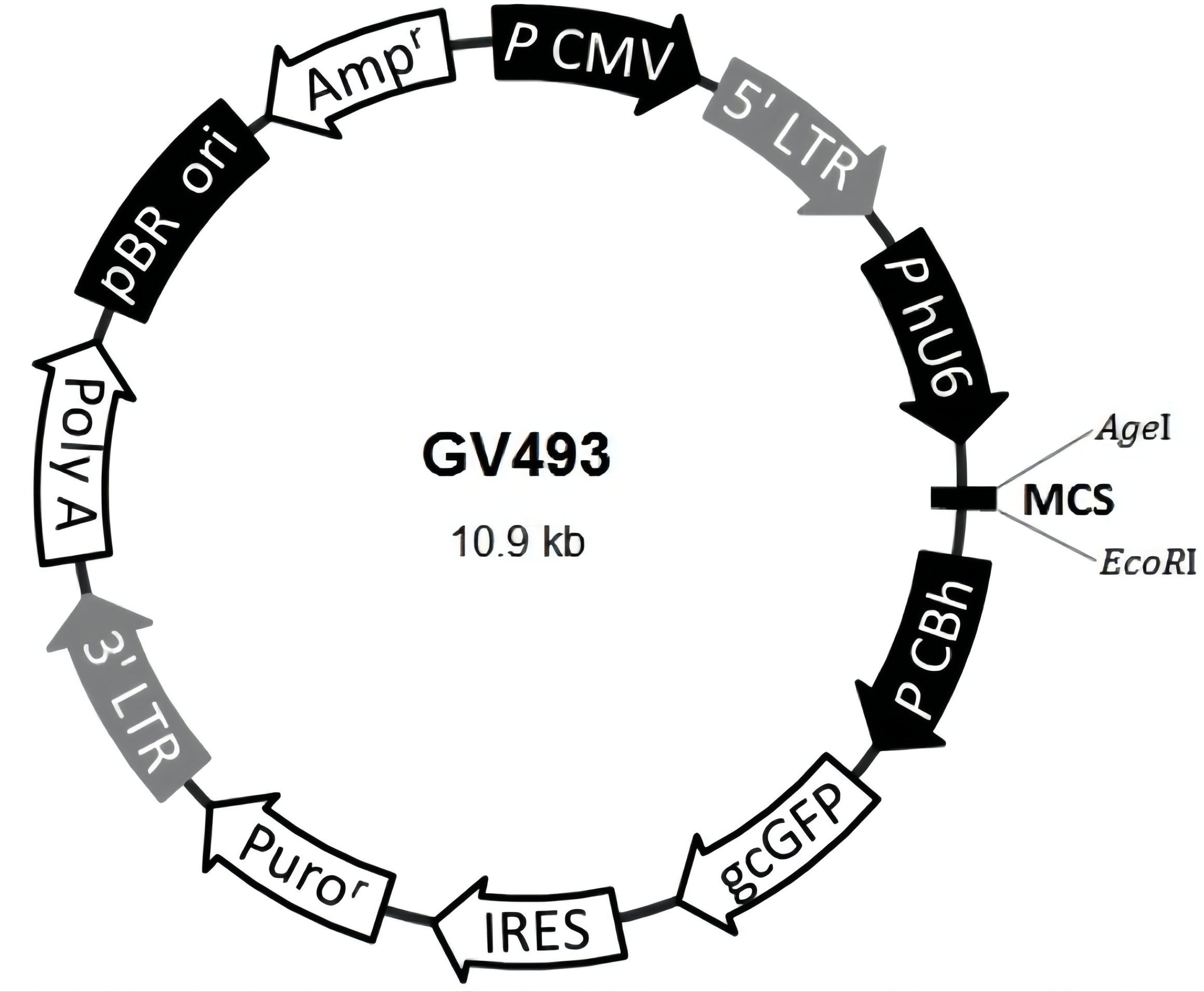

Supplement: Supplementary file 2 — High Resolution Image (TIF 370 KB) [file 12035_2021_2619_MOESM1_ESM.tif]

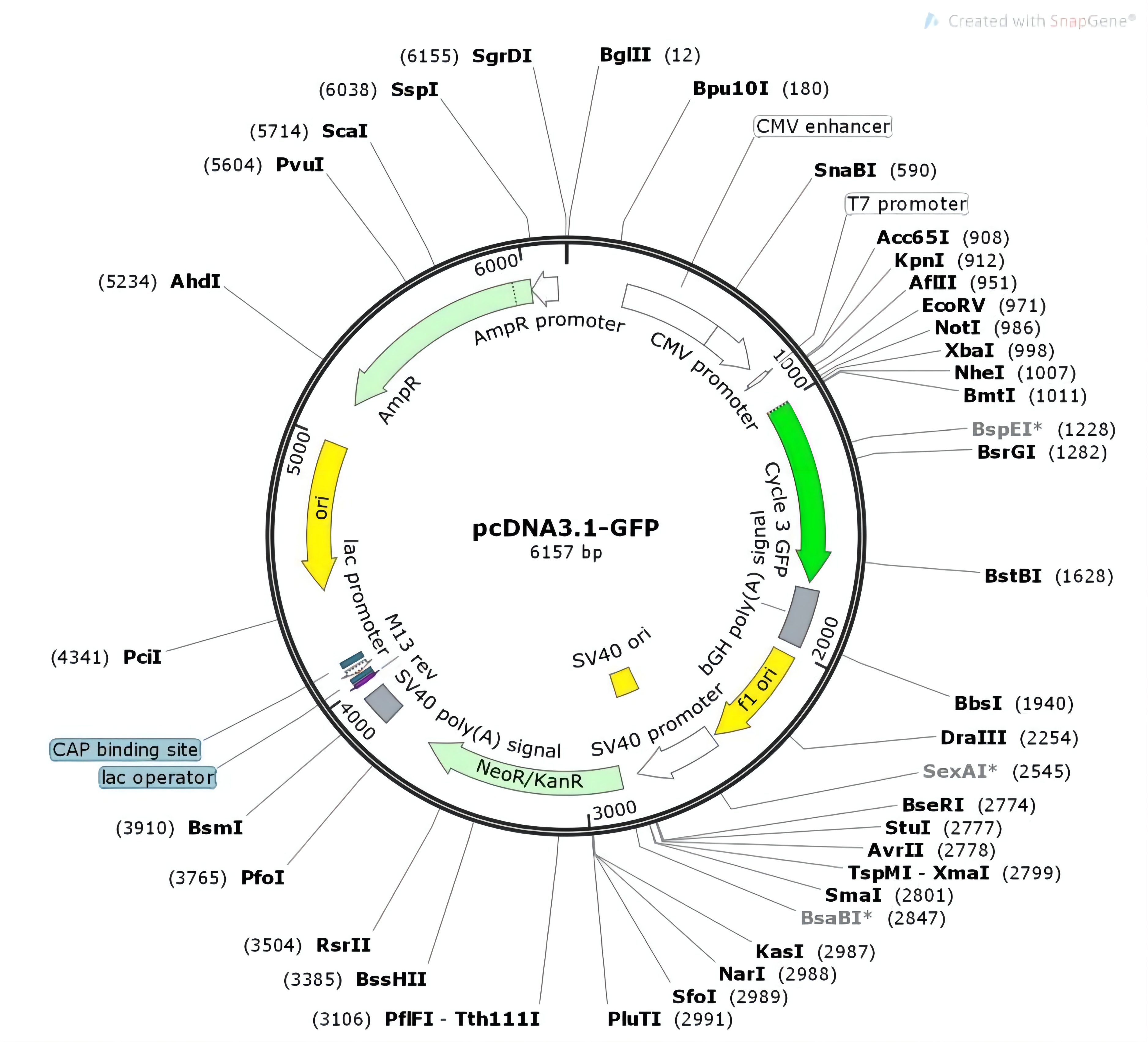

Supplement: Supplementary file 4 — High Resolution Image (TIF 1337 KB) [file 12035_2021_2619_MOESM2_ESM.tif]

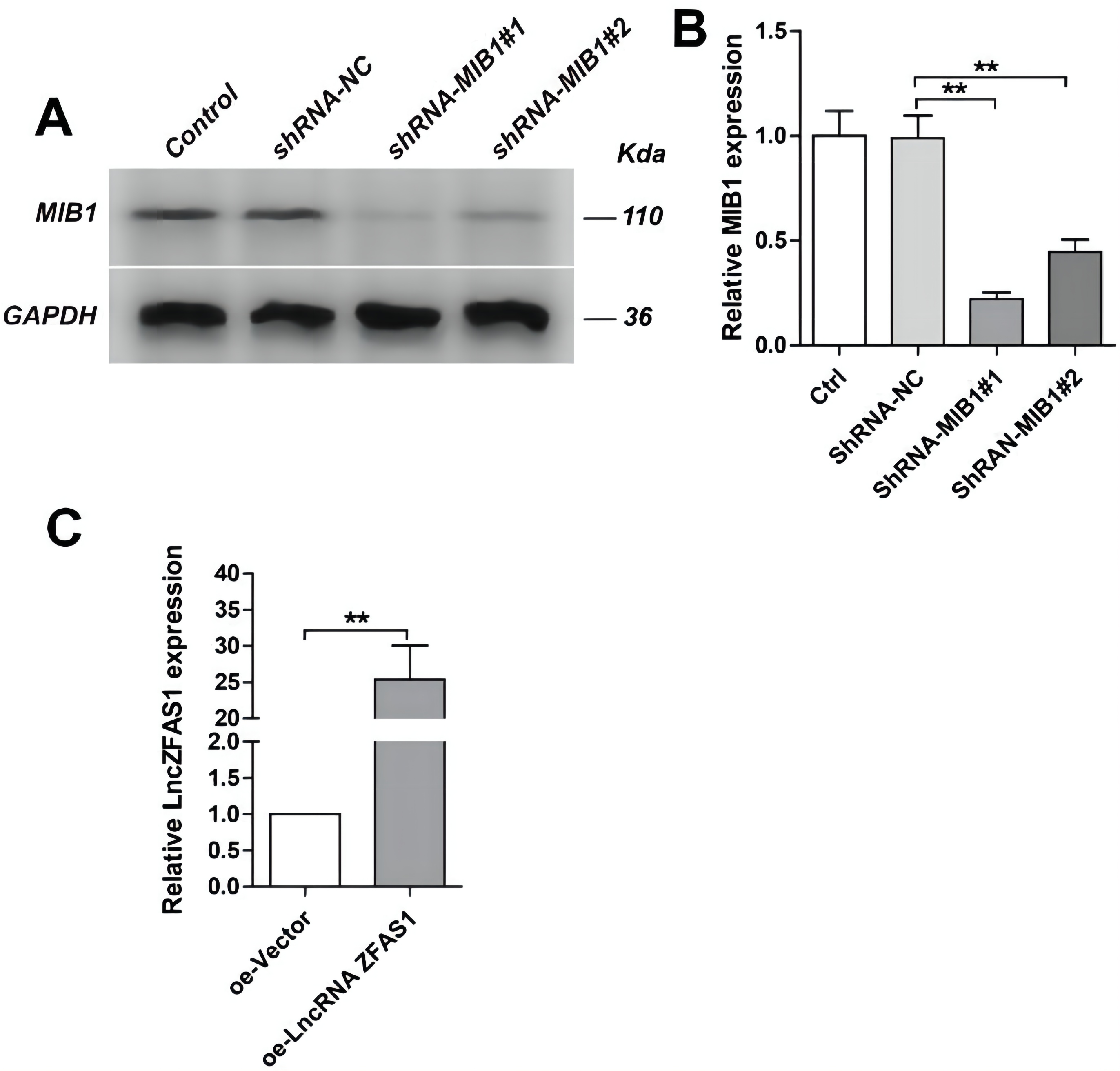

Supplement: Supplementary file 6 — High Resolution Image (TIF 500 KB) [file 12035_2021_2619_MOESM3_ESM.tif]

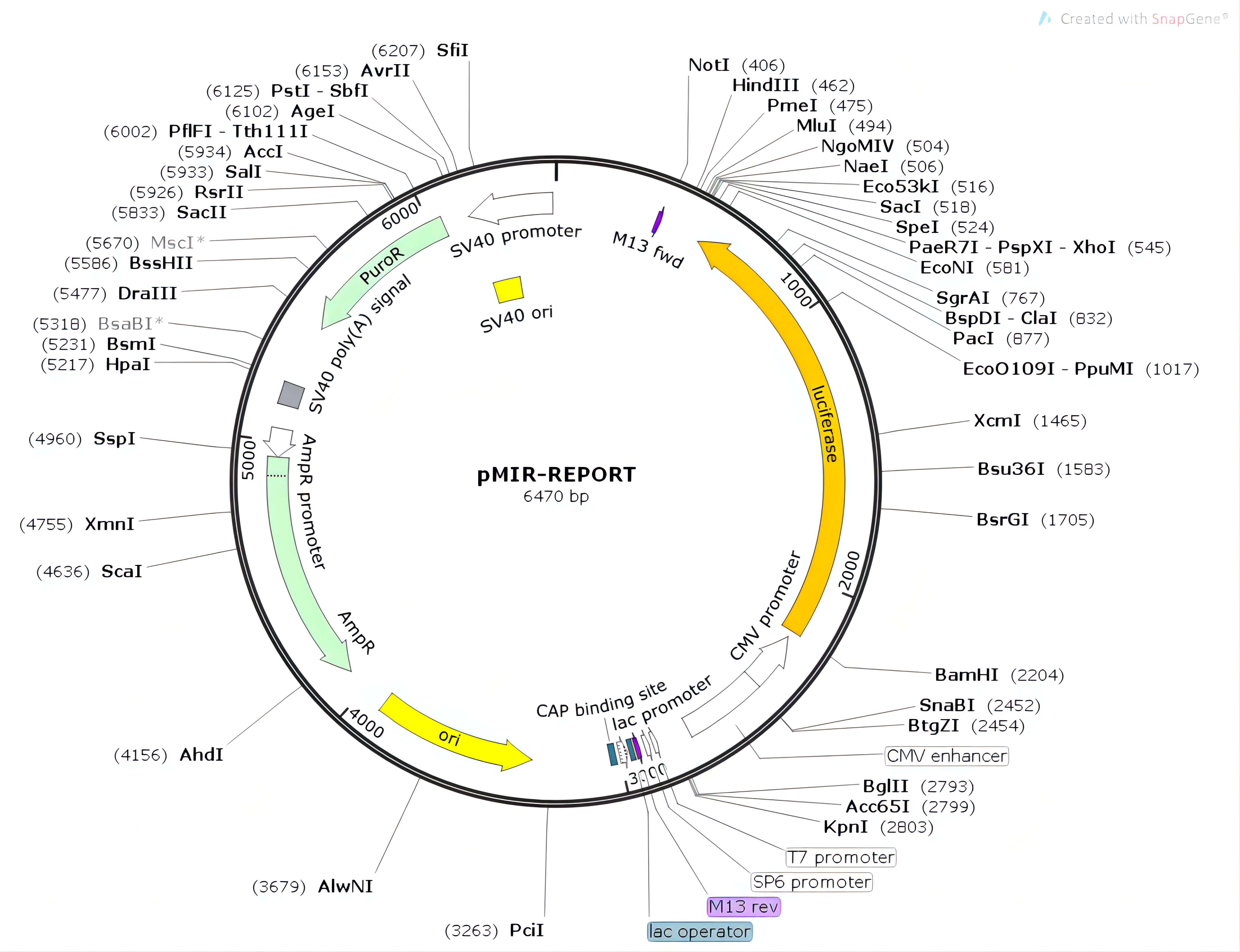

Supplement: Supplementary file 8 — High Resolution Image (TIF 4570 KB) [file 12035_2021_2619_MOESM4_ESM.tif]
